# Oocyte activation deficiency and assisted oocyte activation: mechanisms, obstacles and prospects for clinical application

**DOI:** 10.1093/hropen/hoac003

**Published:** 2022-02-07

**Authors:** Junaid Kashir, Durga Ganesh, Celine Jones, Kevin Coward

**Affiliations:** College of Medicine, Alfaisal University, Riyadh, Kingdom of Saudi Arabia; Department of Comparative Medicine, King Faisal Specialist Hospital and Research Centre, Riyadh, Kingdom of Saudi Arabia; Nuffield Department of Women’s & Reproductive Health, University of Oxford, Level 3, Women’s Centre, John Radcliffe Hospital, Oxford, UK; David Geffen School of Medicine, University of California—Los Angeles, Los Angeles, CA, USA; Nuffield Department of Women’s & Reproductive Health, University of Oxford, Level 3, Women’s Centre, John Radcliffe Hospital, Oxford, UK; Nuffield Department of Women’s & Reproductive Health, University of Oxford, Level 3, Women’s Centre, John Radcliffe Hospital, Oxford, UK

**Keywords:** assisted oocyte activation (AOA), oocyte activation, calcium ionophores, ICSI, phospholipase C zeta (PLCζ), oocyte activation deficiency (OAD), calcium, male infertility, sperm, oocyte

## Abstract

**BACKGROUND:**

Oocyte activation deficiency (OAD) is attributed to the majority of cases underlying failure of ICSI cycles, the standard treatment for male factor infertility. Oocyte activation encompasses a series of concerted events, triggered by sperm-specific phospholipase C zeta (PLCζ), which elicits increases in free cytoplasmic calcium (Ca^2+^) in spatially and temporally specific oscillations. Defects in this specific pattern of Ca^2+^ release are directly attributable to most cases of OAD. Ca^2+^ release can be clinically mediated via assisted oocyte activation (AOA), a combination of mechanical, electrical and/or chemical stimuli which artificially promote an increase in the levels of intra-cytoplasmic Ca^2+^. However, concerns regarding safety and efficacy underlie potential risks that must be addressed before such methods can be safely widely used.

**OBJECTIVE AND RATIONALE:**

Recent advances in current AOA techniques warrant a review of the safety and efficacy of these practices, to determine the extent to which AOA may be implemented in the clinic. Importantly, the primary challenges to obtaining data on the safety and efficacy of AOA must be determined. Such questions require urgent attention before widespread clinical utilization of such protocols can be advocated.

**SEARCH METHODS:**

A literature review was performed using databases including PubMed, Web of Science, Medline, etc. using AOA, OAD, calcium ionophores, ICSI, PLCζ, oocyte activation, failed fertilization and fertilization failure as keywords. Relevant articles published until June 2019 were analysed and included in the review, with an emphasis on studies assessing large-scale efficacy and safety.

**OUTCOMES:**

Contradictory studies on the safety and efficacy of AOA do not yet allow for the establishment of AOA as standard practice in the clinic. Heterogeneity in study methodology, inconsistent sample inclusion criteria, non-standardized outcome assessments, restricted sample size and animal model limitations render AOA strictly experimental. The main scientific concern impeding AOA utilization in the clinic is the non-physiological method of Ca^2+^ release mediated by most AOA agents, coupled with a lack of holistic understanding regarding the physiological mechanism(s) underlying Ca^2+^ release at oocyte activation.

**LIMITATIONS, REASONS FOR CAUTION:**

The number of studies with clinical relevance using AOA remains significantly low. A much wider range of studies examining outcomes using multiple AOA agents are required.

**WIDER IMPLICATIONS:**

In addition to addressing the five main challenges of studies assessing AOA safety and efficacy, more standardized, large-scale, multi-centre studies of AOA, as well as long-term follow-up studies of children born from AOA, would provide evidence for establishing AOA as a treatment for infertility. The delivery of an activating agent that can more accurately recapitulate physiological fertilization, such as recombinant PLCζ, is a promising prospect for the future of AOA. Further to PLCζ, many other avenues of physiological oocyte activation also require urgent investigation to assess other potential physiological avenues of AOA.

**STUDY FUNDING/COMPETING INTERESTS:**

D.G. was supported by Stanford University’s Bing Overseas Study Program. J.K. was supported by a Healthcare Research Fellowship Award (HF-14-16) made by Health and Care Research Wales (HCRW), alongside a National Science, Technology, and Innovation plan (NSTIP) project grant (15-MED4186-20) awarded by the King Abdulaziz City for Science and Technology (KACST). The authors have no competing interests to declare.

WHAT DOES THIS MEAN FOR PATIENTS?

At fertilization, oocyte activation is triggered by sperm-specific phospholipase C zeta (PLCζ) by releasing calcium in specific patterns within the oocyte. A deficiency in this process underlies most cases of fertilization failure in mammals. This process of calcium release can be clinically mimicked via assisted oocyte activation (AOA), involving a combination of mechanical, electrical and/or chemical stimuli.

Recent advances in AOA techniques warrant a review of the safety and efficacy of these practices and of how AOA may be clinically implemented in the clinic. Herein, following a detailed literature review examining studies assessing large-scale efficacy and safety, the main concern impeding clinical AOA implementation is its non-physiological nature, coupled with a lack of holistic understanding of physiological mechanism(s) underlying calcium release at fertilization.

We find that numerous questions require urgent attention before widespread clinical utilization of such protocols can be advocated. We hope that this article will be able to aid the burgeoning number of researchers investigating the clinical efficacy of such methodology in further refining the practice until large-scale utilization can be achieved and accepted.

## Introduction

Of all cases of infertility, 30–50% can be attributed to a male causative factor (Kumar and Singh, 2015; Leaver, 2016), while about 30% of all cases of infertility cannot currently be explained (Ray *et al.*, 2012). In both instances, ICSI (whereby a single sperm is injected directly into the oocyte) represents a standard mode of treatment (Palermo *et al.*, 2015; Rubino *et al.*, 2016; Simopoulou *et al.*, 2016; Palermo *et al.*, 2017; Borges *et al.*, 2020b). ICSI itself is just one of a suite of laboratory techniques designed to treat various forms of infertility, collectively termed ART. However, ICSI usage increased from 15.4% to 66.9% between 1996 and 2012, even though ICSI is not universally recommended for normospermic men (Boulet *et al.*, 2015; Sustar *et al.*, 2019; Ten *et al.*, 2019). ICSI yields fertilization rates of 70–80%, but failed fertilization following ICSI, sometimes recurrent over repeated cycles and miscarriage remain a difficult reality for many couples (Montag *et al.*, 2012; Yoon *et al.*, 2013; D’Haeseleer *et al.*, 2014; Nikiforaki *et al.*, 2016; Karabulut *et al.*, 2018; Koot *et al.*, 2019; Rumbold *et al.*, 2019; Duran‐Retamal *et al.*, 2020). Worryingly, numerous indications now suggest an increased incidence of birth defects in ART babies compared to naturally conceived counterparts (Zhao *et al.*, 2020).

Total fertilization failure (TFF) occurs in 1–5% of ICSI cases (Kyono *et al.*, 2012; Montag *et al.*, 2012; Vanden Meerschaut *et al.*, 2014b; Kashir *et al.*, 2018; Basirat *et al.*, 2019; Bassiri *et al.*, 2020). Moreover, associations have been identified between embryo grading and congenital malformations, endocrine profile changes and deficient hearing in ICSI-conceived babies (Abel *et al.*, 2019; Belva *et al.*, 2019; Yasemin Sert *et al.*, 2019), while age, antimullerian hormone levels and antral follicle counts are associated with live birth rates following ICSI, with other hormones currently under investigation (Metello *et al.*, 2019; Zheng *et al.*, 2019; Song *et al.*, 2020; Tarín *et al.*, 2020; Tiegs *et al.*, 2020; Wang *et al.*, 2020; Ye *et al.*, 2020; Zanetti *et al.*, 2020). The use of ART however does not seem linked to an increase in the risk of autism spectrum disorder or preeclampsia (Diop *et al.*, 2019; Kennedy *et al.*, 2019). Only frozen embryo transfer was associated with a small yet statistically significant increased risk for childhood cancer (Hargreave *et al.*, 2019), and the cognitive, behavioural and school performance of children born from IVF versus ICSI remain comparable (Heineman *et al.*, 2019; Norrman *et al.*, 2020).

Various factors account for ICSI failure, including unexplained non-male factor infertility (Gennarelli *et al.*, 2019). However, a failure or defect in a series of concerted events at fertilization, collectively termed oocyte activation deficiency (OAD), whether sperm- or oocyte-borne, is the main cause for TFF (Tesarik *et al.*, 2002; Vanden Meerschaut *et al.*, 2014b; Deemeh *et al.*, 2015; Capalbo *et al.*, 2016; Tosti and Ménézo, 2016; Karabulut *et al.*, 2018; Kashir *et al.*, 2018), resulting in the inability of mature oocytes to undergo activation and complete fertilization by sperm. This is thought to be directly responsible for 40% of ICSI failures (Heindryckx *et al.*, 2008; Yeste *et al.*, 2016a), with perhaps a higher level attributable in an indirect manner (Sang *et al.*, 2018; Yang *et al.*, 2019; Kashir, 2020; Kashir *et al.*, 2020b).

Perhaps due to the increasing proportion of ICSI cycles, the increasing rates of TFF and OAD are to be expected (Montag *et al.*, 2012; Santella and Dale, 2015; Aydinuraz *et al.*, 2016; Ebner and Montag, 2016; Bonte *et al.*, 2019). Significant efforts have focussed on elucidating the molecular mechanisms underlying both oocyte activation and fertilization failure, and the clinical methodologies used to rectify cases of OAD, termed assisted oocyte activation (AOA). However, the safety and efficacy of such methods remains controversial, with no clear indication as to whether such protocols should be applied or not within the clinic. Herein, we review recent findings regarding AOA efficacy and safety, exploring the major obstacles preventing widespread use of AOA in clinical practice, in line with recent clinical evaluation on the utilization of AOA. We also briefly examine the molecular mechanisms underlying oocyte activation and posit potential alternatives to current strategies for AOA to improve the efficacy of such treatments, with perhaps an improvement in ART success rates.

## Methods

This article is based on a critical review of literature on peer-reviewed article indexing databases including PubMed, Scopus and Medline, using AOA, OAD, calcium ionophores, ICSI, phospholipase c zeta (PLCζ), oocyte activation, failed fertilization and fertilization failure as keywords.

## Physiological mechanism of oocyte activation

**Figure 1.**
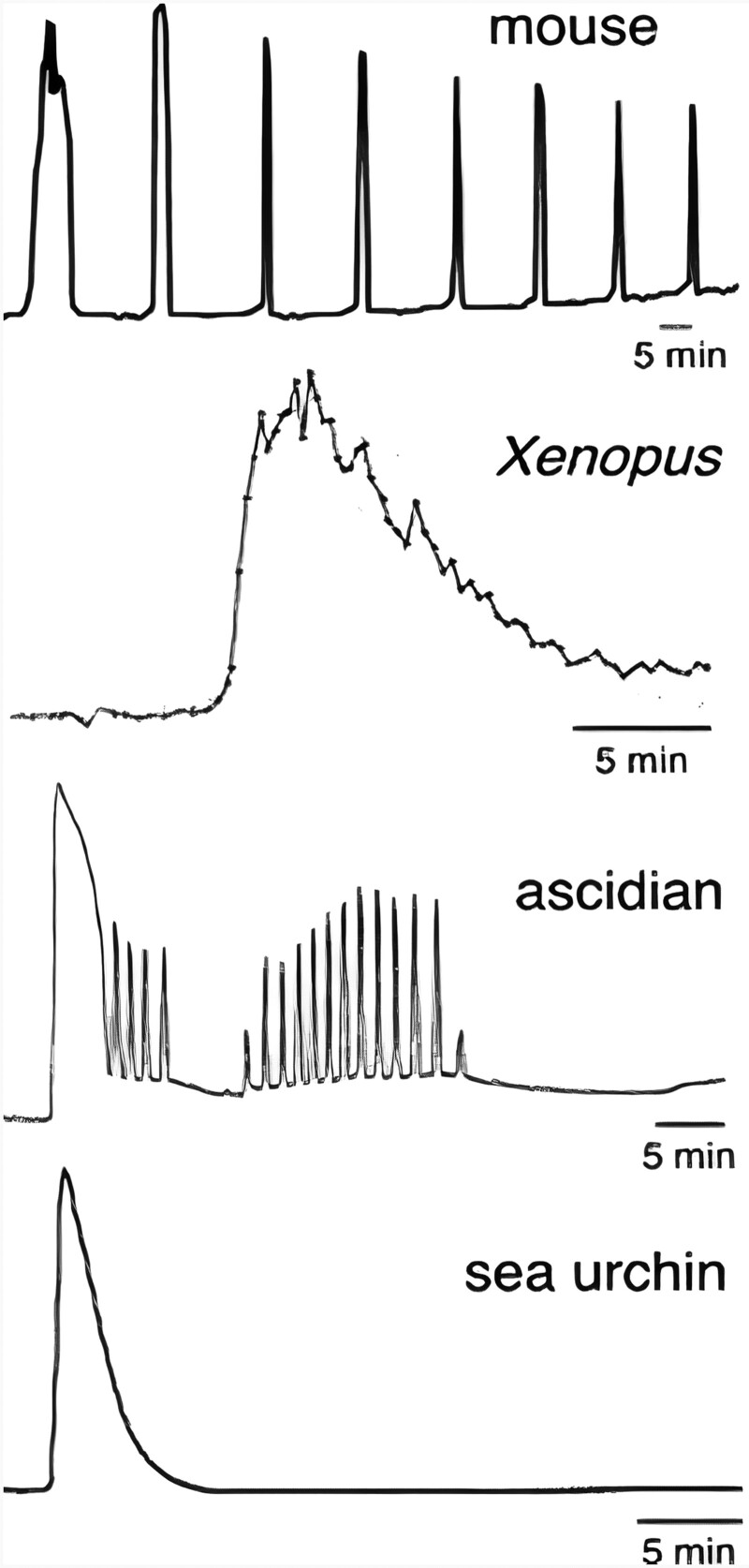
**Representative Ca^2+^ responses at fertilization in eggs/oocytes of several species.** Figure reproduced from Miyazaki (2006) with permission.

Oocyte activation is a spatially and temporally orchestrated process (Xu and Yang, 2017), resulting in established endpoints including resumption of meiosis II (MII), second polar body (2PB) extrusion, cortical granule exocytosis and cytoskeletal rearrangements (Hosseini *et al.*, 2008; Tosti and Ménézo, 2016; Kashir *et al.*, 2018; Sha *et al.*, 2019). In mammals, these events are a collective culmination of temporally-mediated cytoplasmic calcium (Ca^2+^) levels, initiated in an inositol 1,4,5-trisphosphate receptor-dependent manner from Ca^2+^ stores such as the endoplasmic reticulum (ER) (Alberio *et al.*, 2000; Yeste *et al*., 2016a). Observed in all species till date, the spatial and temporal pattern of the Ca^2+^ release is species-specific in amplitude, frequency and number (Fig. 1) and in molecular modulation (Kashir *et al.*, 2018). Other components at the transcriptional and translational level certainly play a role (Dorfeshan *et al.*, 2019; Rong *et al.*, 2019; Sha *et al.*, 2019; Zhang *et al.*, 2019; Kang *et al.*, 2020; Sacha *et al.*, 2020; Sha *et al.*, 2020; Zhang *et al.*, 2020), but we focus herein on the wave-like Ca^2+^ diffusion that is integral to oocyte activation.

### Ca^2+^ at oocyte activation

**Figure 2.**
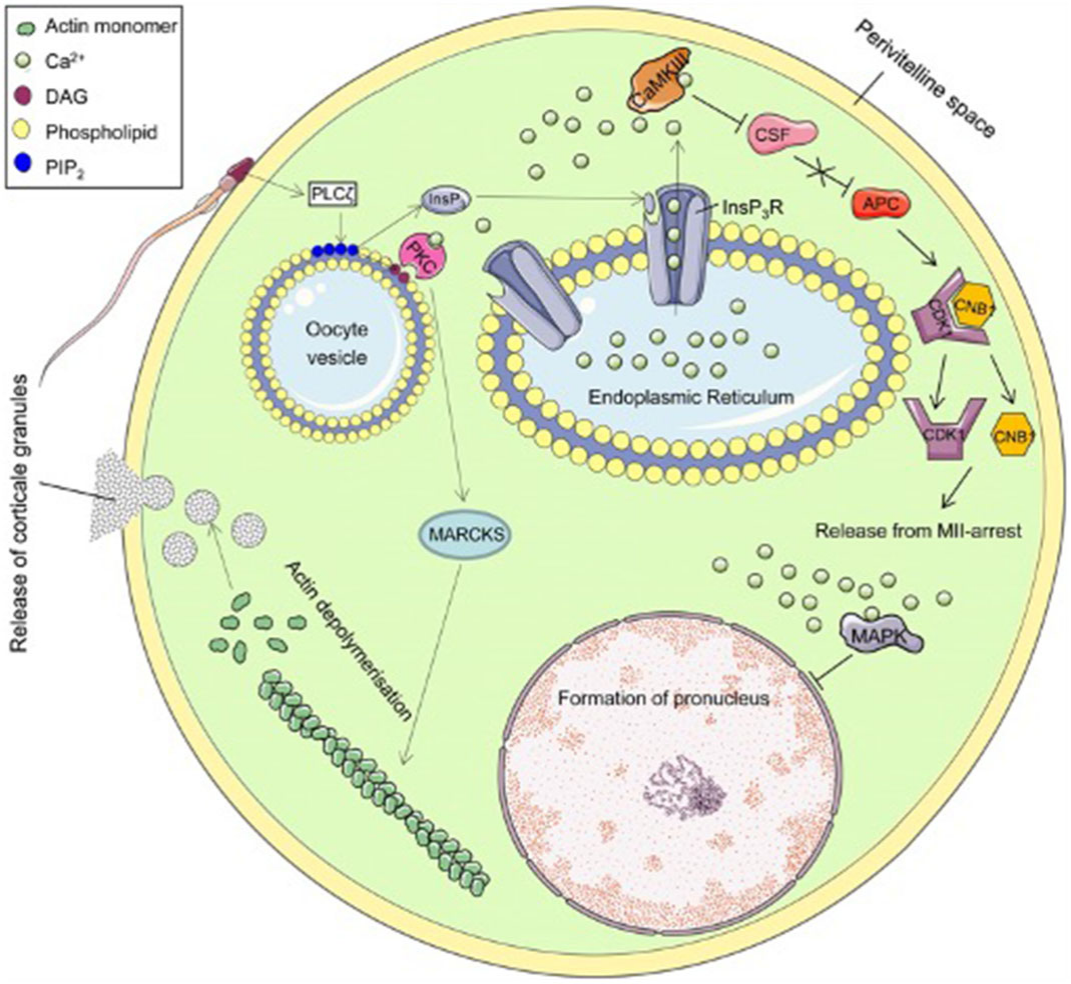
**Schematic summary of the proposed mechanism underlying Ca^2+^ release at oocyte activation.** The fertilizing sperm triggers Ca^2+^ following delivery of sperm-specific phospholipase C zeta (PLCζ) to the oolemma during or following oocyte-sperm membrane fusion. PLCζ interacts with an as yet unknown oocyte-borne factor(s), facilitating hydrolysis of PIP_2_ into DAG and InsP_3_, which subsequently triggers Ca^2+^ release from intracellular stores, alleviating the MII-arrest. The proposed mechanism mediates cortical granules exocytosis, MAPK deactivation and subsequent pronuclei formation and CaMKII activation, inhibiting CSF (Emi2) and liberating APC. This reduces levels of Cyclin B1 in the maturation-promoting factor (MPF) complex comprising CDK1 and Cyclin B1, which inactivates MPF, releasing the oocyte from MII-arrest. APC, anaphase-promoting complex/cyclosome; CaM/CaMKII, calcium/calmodulin-dependent protein kinase II; CSF, cytostatic factor; CNB1, cyclin B1; CDK1, cyclin-dependent kinase 1; DAG, diacylglycerol; InsP_3_, inositol 1,4,5-trisphosphate; InsP_3_R, InsP_3_ receptor; MAPK, mitogen-activated protein kinase; PIP_2_, phosphatidylinositol 4,5-bisphosphate; PKC, protein kinase C. Schematic reproduced with permission from Yeste *et al.* (2016b).

Ca^2+^-sensitive dyes have revealed in non-mammalian species that activation is triggered by a single large transient increase in Ca^2+^ (Horner and Wolfner, 2008). However, mammalian oocytes undergo a series of these Ca^2+^ transients, defined as oscillations (Publicover *et al.*, 2007; Swann and Yu, 2008). Importantly, the specific patterns of Ca^2+^ release in terms of amplitude, duration and frequency over time seem largely species-specific in all species studied to date (Miyazaki *et al.*, 1993; Jones *et al.*, 1998b; Stricker, 1999; Ducibella *et al.*, 2002, 2006; Ducibella and Fissore, 2008). Ca^2+^ oscillations in mammalian oocytes are a direct consequence of cytosolic inositol trisphosphate (IP_3_), indicating that this signalling cascade initiates with hydrolysis of phosphatidylinositol 4,5-bisphosphate (PIP_2_) (Fig. 2) (Parrington, 2001; Swann *et al.*, 2006; Whitaker, 2006; Parrington *et al.*, 2007; Swann and Yu, 2008). Microinjecting Ca^2+^ ions triggers mouse blastocyst development (Fulton and Whittingham, 1978; Swann and Yu, 2008), while blocking, down-regulating or reducing levels of IP_3_-Rs inhibits Ca^2+^ oscillations and oocyte activation in mouse and hamster oocytes (Miyazaki *et al.*, 1993; Brind *et al.*, 2000; Jellerette *et al.*, 2000; Xu *et al.*, 2003). Cytosolic IP_3_ peaks are also observed during fertilization in mammalian oocytes (Swann and Yu, 2008).

Mammalian Ca^2+^ oscillations alleviate MII arrest through cyclin B1 proteolysis, mediated by ubiquitin or proteasome activation (Fig. 2) (Miyazaki and Ito, 2006). Ca^2+^–calmodulin association activates calmodulin-dependent kinase II (CaMKII) (Miyazaki and Ito, 2006) in a repetitive manner that occurs coincident with each Ca^2+^ peak in fertilizing mouse oocytes, resulting in Cyclin B1 polyubiquitination by the anaphase promoting complex/cyclosome (APC/C), a E3 ubiquitin ligase (Swann and Lai, 2016). This cascade is prevented in unfertilized oocytes by cytostatic factor (CSF), maintaining MII arrest (Hyslop *et al.*, 2004; Jones, 2004; Miyazaki and Ito, 2006). Upon fertilization, CaMKII inhibits CSF components (Hyslop *et al.*, 2004). Persistent Ca^2+^ oscillations also contribute to pronuclear formation via reduction of mitogen-associated protein kinase activity (Ducibella *et al.*, 2002; Miyazaki and Ito, 2006).

Such is the importance of Ca^2+^ profiles at fertilization that the frequency and amplitude of Ca^2+^ oscillations affected early embryo protein profiles in mice (Ducibella *et al.*, 2002) and embryonic development in rabbits, determining transplantation rates of four-cell embryos (Swann and Ozil, 1994; Miyazaki and Ito, 2006). Oocyte activation events are also temporally and spatially sensitive to specific Ca^2+^ oscillation profiles in mammals in a chronological manner, with early events such as cortical granule exocytosis requiring fewer oscillations than later events such as the alleviation of MII arrest (Malcuit *et al.*, 2006; Stitzel and Seydoux, 2007). Furthermore, the number of Ca^2+^ pulses required to complete oocyte activation is greater than the number required to initiate exit from MII arrest (Ducibella *et al.*, 2002; Krauchunas and Wolfner, 2013). Thus, this degree of sensitivity and potential downstream effect on embryogenesis necessitated the elucidation of the causative stimulant that initiates these essential patterns of Ca^2+^ release.

The precise mechanisms underlying Ca^2+^ oscillations in mammals have been subject to much debate, particularly regarding the roles played by both gametes. Three predominant models were hypothesized: (i) the Ca^2+^ conduit model (Jaffe, 1983, 1991), (ii) the membrane receptor model (Jaffe, 1990; Schultz and Kopf, 1995; Evans and Kopf, 1998; Parrington *et al.*, 2007) and (iii) the soluble sperm factor model (Swann *et al.*, 2006; Parrington *et al.*, 2007; Saunders *et al.*, 2007). Data obtained for one or more species can be interpreted as supporting each of these models as being the causative theory behind oocyte activation, which are more thoroughly reviewed elsewhere (Stricker, 1999; Kashir *et al.*, 2013a). However, significant evidence supports the theory of sperm factor mediation within mammals and other taxa (Jones *et al.*, 1998b; Swann *et al.*, 2006; Whitaker, 2006; Parrington *et al.*, 2007; Iwao, 2012).

The ‘sperm-factor’ model suggests that oocyte activation is triggered by a soluble factor released from the sperm into the oocyte during, or immediately following, gamete fusion (Lawrence *et al.*, 1997). Injecting sperm extracts into the eggs of a variety of species resulted in successful Ca^2+^ release and oocyte activation (Swann, 1990; Kyozuka *et al.*, 1998; Stricker, 1999; Dong *et al.*, 2000; Coward *et al.*, 2003) suggesting that similar sperm-based oocyte activation mechanisms exist throughout a wide spectra of (at least invertebrate) species. The soluble sperm factor responsible for initiating activation in mammalian oocytes seems sperm-specific, as injection of other somatic cells into oocytes does not cause Ca^2+^ transients (Swann, 1990; Wu *et al.*, 1997; Kashir *et al.*, 2013a), while ICSI also successfully results in oocyte activation and fertilization.

### Regulation of calcium stores

While the major source of Ca^2+^ release at fertilization is the ER, extracellular Ca^2+^ influx is required to maintain these Ca^2+^ oscillations. Indeed, depletion of extracellular Ca^2+^ reduces the frequency of or completely ceases Ca^2+^ oscillations (Igusa and Miyazaki, 1983; Kline and Kline, 1992a; Takahashi *et al.*, 2013). Indeed, Ca^2+^ influx also seems to play a crucial role in determining the intervals between Ca^2+^ transients (Takahashi *et al.*, 2013). A candidate phenomenon underlying Ca^2+^ store regulation has been a mechanism termed store operated Ca^2+^ entry (SOCE), which maintains intracellular Ca^2+^ homeostasis from the extracellular milieu within at least somatic cells (Putney, 1986; Miao and Williams, 2012). Major regulators of SOCE seem to be the STIM proteins (STIM1/STIM2), transmembrane ER proteins that bind Ca^2+^ (Williams *et al.*, 2001; Roos *et al.*, 2005; Cai, 2007; Hewavitharana *et al.*, 2007; Miao and Williams, 2012).

The sensitivity of STIM2 to even minor decreases in Ca^2+^ levels within the ER lumen allows it to stabilize basal cytosolic and ER Ca^2+^ levels under non-stimulated conditions (Brandman *et al.*, 2007). STIM1, however, can only be activated by larger decreases in ER luminal Ca^2+^ following large-scale Ca^2+^ release (such as at oocyte activation) (Miao and Williams, 2012). In somatic cells, plummeting ER Ca^2+^ causes oligomerization and redistribution of STIM1, activating STIM1 (Liou *et al.*, 2005; Smyth *et al.*, 2008), which then signal ORAI proteins, stimulating transport of extracellular Ca^2+^ into the cytosol (Zhang *et al.*, 2005; Feske *et al.*, 2006; Vig *et al.*, 2006). Sarcoplasmic/ER Ca^2+^ ATPase pumps can then transport Ca^2+^ back into the ER, replenishing cellular stores (Miao and Williams, 2012).

Considering that mammalian oocytes express both STIM1 and ORAI1 (Gómez-Fernández *et al.*, 2009; Koh *et al.*, 2009), while mouse oocytes also express STIM2, this would suggest a similar mechanism for Ca^2+^ store regulation in oocytes (Miao and Williams, 2012). However, recent data have indicated that female mice lacking one or both STIM proteins remained fertile, with oocytes exhibiting normal patterns of Ca^2+^ release post-fertilization, as well as ER Ca^2+^ stores or Ca^2+^ influx following depletion (Bernhardt *et al.*, 2017). Similar observations were also made with oocytes from mice lacking ORAI (Bernhardt *et al.*, 2017). Such data perhaps indicate that the STIM1/STIM2/ORAI mechanisms do not play a major role at least within mouse oocytes.

Indeed, neither known SOCE blockers nor the expression of STIM1/ORAI inhibitory protein fragments affect the Ca^2+^ oscillation frequency or influx rate (Takahashi *et al.*, 2013). Intriguingly, however, Bernhardt *et al.* (2017) also indicated that fertilization-associated patterns of Ca^2+^ release were impaired by NS8593, a TRPM7-specific inhibitor. Oocytes depleted of both TRPM7 and CaV3.2 terminate oscillations prematurely, with a concurrent delay in resumption of oscillations, strongly indicating a collective action of multiple factors in maintaining the majority of Ca^2+^ influx following fertilization (Bernhardt *et al.*, 2018). Collectively, such findings suggest that CaV3.2 and TRPM7 serve as essential mediators of Ca^2+^ influx following fertilization, at least within mice (Stein *et al.*, 2020). Double knock out mice depleted of both TRPV3 and CaV3.2 were subfertile, with reduced oocyte Ca^2+^ stores. Furthermore, the number of double knock-out oocytes exhibiting Ca^2+^ release was significantly lower that WT at fertilization, and oscillations were also of reduced frequency (Mehregan *et al.*, 2021). Collectively such studies indicate that the collective action of TRPV3 and CaV3.2 is required for both initiation and specific profiles (amplitude, frequency and longevity) of Ca^2+^ oscillations during fertilization, at least within mammals (Mehregan *et al.*, 2021).

While such suggestions may be true for mice, this may not be the entire picture for all mammals. Indeed, evidence suggests that in the pig, Ca^2+^ oscillations are indeed supported by SOCE, with Ca^2+^ release accompanied by repeated interactions between STIM1 and ORAI, while a STIM1 puncta formation inhibitor (ML-9) blocks SOCE, also disrupting Ca^2+^ oscillations in fertilized oocytes (Zhang *et al.*, 2018). Lysosomes may also exhibit some degree of Ca^2+^ uptake mechanisms. However, the exact underlying mechanisms remain unknown (Lloyd-Evans and Waller-Evans, 2020). Mitochondria can also uptake Ca^2+^ through the mitochondrial Ca^2+^ uniporter, and in the presence of high cytoplasmic Ca^2+^, through voltage-dependent anion channels (Romero-Garcia and Prado-Garcia, 2019; Stein *et al.*, 2020).

### PLCζ, oocyte activation and male infertility

Numerous studies have attempted to identify the most physiologically relevant candidate for the sperm factor in mammals, initially suggesting that a 33 kDa oscillogen initiated Ca^2+^ oscillations (Parrington *et al.*, 1996), yet recombinant versions were unable to elicit Ca^2+^ oscillations in mouse oocytes (Wolosker *et al.*, 1998; Parrington *et al.*, 1999; Swann *et al.*, 2004). Subsequently, the truncated form of the c-kit receptor, tr-kit, was proposed to induce parthenogenetic mouse oocyte activation (Sette *et al.*, 1997, 2002) purportedly via activation of phospholipase C gamma-1 (PLCγ1) through phosphorylation by a Src-like Kinase Fyn (Sette *et al.*, 1998). However, independent efforts have thus far failed to replicate such results, or emulate these in humans (Kashir *et al.*, 2014).

Another more recent proposal is the post-acrosomal sheath WW domain-binding protein (PAWP), microinjection of which into porcine, bovine, macaque and *Xenopus* eggs resulted in pronuclear formation (Wu *et al.*, 2007). PAWP injection also caused a Ca^2+^ increase when injected into *Xenopus* eggs but did not appear to mimic the single large Ca^2+^ wave normally seen at fertilization in such eggs (Aarabi *et al.*, 2009; 2014a,b). However, despite earlier preliminary studies (Aarabi *et al.*, 2014a,b), repeated independent experiments could not demonstrate the ability of recombinant mouse and human PAWP to elicit any detectable Ca^2+^ release when microinjected into mouse oocytes (Kashir *et al.*, 2014; Nomikos *et al.*, 2015). However, when one considers that sperm-induced Ca^2+^ oscillations seem to be caused by activation of the inositol 1,4,5-trisphosphate (InsP_3_) signalling pathway (Miyazaki *et al.*, 1992), this suggests that the sperm factor might itself be a phospholipase C (PLC) isoform (Jones *et al.*, 1998a).

Of the known mammalian phosphoinositide (PI)-specific PLC isozymes at the time (Kelley *et al.*, 2001; Rhee, 2001; Song *et al.*, 2001; Hwang *et al.*, 2005; Nakahara *et al.*, 2005; Zhou *et al.*, 2005), very few were able to successfully and physiologically result in successful oocyte activation (Yeste *et al.*, 2016a; Kashir *et al.*, 2018). Indeed, while several PLCs exert specific roles at fertilization within both gametes (PLC delta 4 in the acrosome reaction, or PLC beta 1 in regulating calcium dynamics in the oocyte) (Fukami *et al.*, 2001, 2003; Igarashi *et al.*, 2007), most PLC isoforms do not seem directly involved in oocyte activation, failing to elicit Ca^2+^ release upon injection into mouse oocytes (Kashir, 2020).

It was not until mouse express sequence tag (EST) databases were used that a testis-specific PLC was described, termed PLCzeta (PLCζ), a ∼74 kDa protein in mice and ∼70 kDa in humans (Cox *et al.*, 2002; Saunders *et al.*, 2002). Immunodepletion of PLCζ from sperm extracts diminished Ca^2+^ release following injection into mouse oocytes or sea urchin egg homogenates (Saunders *et al.*, 2002), while recombinant PLCζ injections in mouse oocytes elicited fertilization-like Ca^2+^ oscillations, supporting blastocyst development (Saunders *et al.*, 2002; Kouchi *et al.*, 2004). Finally, disruption of PLCζ expression in mice testes through RNA interference exhibited sperm that induced prematurely ending Ca^2+^ oscillations. While these mice were not infertile, mating experiments yielded significantly reduced litter sizes (Knott *et al.*, 2005). Finally, two papers (Hachem *et al.*, 2017; Nozawa *et al.*, 2018) recently reported the creation of transgenic knockout mouse models of PLCζ, both concluding (albeit with caveats as discussed later herein) that PLCζ is the primary physiological stimulus of Ca^2+^ oscillations at fertilization.

PLCζ is much more potent in mouse oocytes compared to other PLCs (Swann *et al.*, 2006, 2007; Swann and Lai, 2016). Although further details regulating the molecular mechanisms underlying PLCζ mechanistic action is urgently required, PLCζ is thought to target PIP_2_-containing cytoplasmic vesicular lipids within the oocyte (Yu *et al.*, 2012) to form IP_3_, leading to subsequent Ca^2+^ release from stores such as the ER in an IP_3_R-dependent manner (Fig. 2) (Swann and Lai, 2016; Swann, 2020). PLCs usually produce IP_3_ through PIP_2_ that are present almost exclusively in membranes, suggesting that PLCζ should target the oocyte plasma membrane, where cells normally contain the bulk of their PIP_2_ (Halet *et al.*, 2002; Yu *et al.*, 2012). However, rather than a decrease of PIP_2_ as would be expected in such a case, an increase in PIP_2_ levels at the plasma membrane is observed following both normal fertilization and PLCζ microinjection (Halet *et al.*, 2002), while conversely, PLCδ1 injection led to a loss of plasma membrane PIP_2_ (Yu *et al.*, 2012; Swann and Lai, 2016). Indeed, the first Ca^2+^ transient during mammalian fertilization initiates from the point of gamete fusion, with subsequent Ca^2+^ release occurring from multiple regions throughout the fertilizing oocyte, implying that the majority of PIP_2_ hydrolysis and InsP_3_ generation occurs within the oolemma (Fig. 2); these assertions supported by numerous experimental and theoretical models (Whitaker and Irvine, 1984; Dupont and Dumollard, 2004; Sanders and Swann, 2016).

PLCζ was observed uniformly distributed throughout the oocyte cytoplasm, not at the plasma membrane (Yoda *et al.*, 2004; Yu *et al.*, 2008), specifically within vesicles no bigger than 1 µm (Yu *et al.*, 2012). This suggests that IP_3_ is generated from an intracellular source of PIP_2_ during fertilization. Indeed, mammalian sperm extracts (containing PLCζ) hydrolyze PIP_2_ in sea urchin egg homogenates, while maximal IP_3_ was generated in those fractions of homogenates that were richest in yolk, vesicles which demonstrably contain PIP_2_ (Snow *et al.*, 1996; Rice *et al.*, 2000). However, as mouse oocytes do not contain yolk, the nature of the observed vesicles remains to be determined (Swann and Lai, 2016).

Several conditions of male infertility (accounting for 19–57% of cases of total infertility) currently remain untreatable following application of ART (Kashir, 2020; Kashir *et al.*, 2010; Saleh *et al.*, 2020), even following ICSI. Indeed, up to 5% of ICSI treatment cycles still fail, which is largely attributed to a defect in oocyte activation (Kashir *et al.*, 2010). Sperm from infertile males which consistently fail to fertilize oocytes following ART (IVF or ICSI) either fail to elicit Ca^2+^ oscillations, or do so abnormally (Yoon *et al.*, 2008; Heytens *et al.*, 2009). Such sperm also exhibited abnormal patterns and levels of PLCζ within the sperm head, suggesting that defects in sperm PLCζ (at both gene and protein levels) may underlie such cases of fertilization failure, particularly considering that such fertilization failure can be ‘rescued’ following concurrent microinjection of infertile human sperm with recombinant PLCζ (Yoon *et al.*, 2008).

However, further to the 1–5% of ICSI cycles expected to experience TFF, even the response from ‘fertile’ males is extremely variable, with ∼20% of human sperm exhibiting only 1 or 2 Ca^2+^ transients upon injection into oocytes (Ferrer-Buitrago *et al.*, 2018a,b), which is unlikely to activate oocytes (Swann, 2020). Furthermore, 10% of such sperm did not elicit any Ca^2+^ release at all, suggesting that the ability of normal human sperm to cause Ca^2+^ signals is likely to be highly variable; such assertions are in line with observations of variable localization patterns and levels of PLCζ in human sperm, and may underlie cases of low fertilization success (a much more common occurrence) in addition to TFF (Kashir *et al.*, 2013b, 2020b; Yelumalai *et al.*, 2015; Yeste *et al.*, 2016b).

Clinically, complete fertilization failure is attributed to defective oocyte activation failure in a sperm-specific manner, more so than any other potential cause (Kashir *et al.*, 2010). Furthermore, an increasing body of evidence is now associating PLCζ defects with not just outright OAD, but also a growing number of male factor conditions affecting sperm DNA integrity, morphology, count and motility, as well as the efficacy of cell cycle resumption rates and resulting embryogenesis (refer to (Kashir, 2020) for details).

## Common methods of AOA

**Figure 3.**
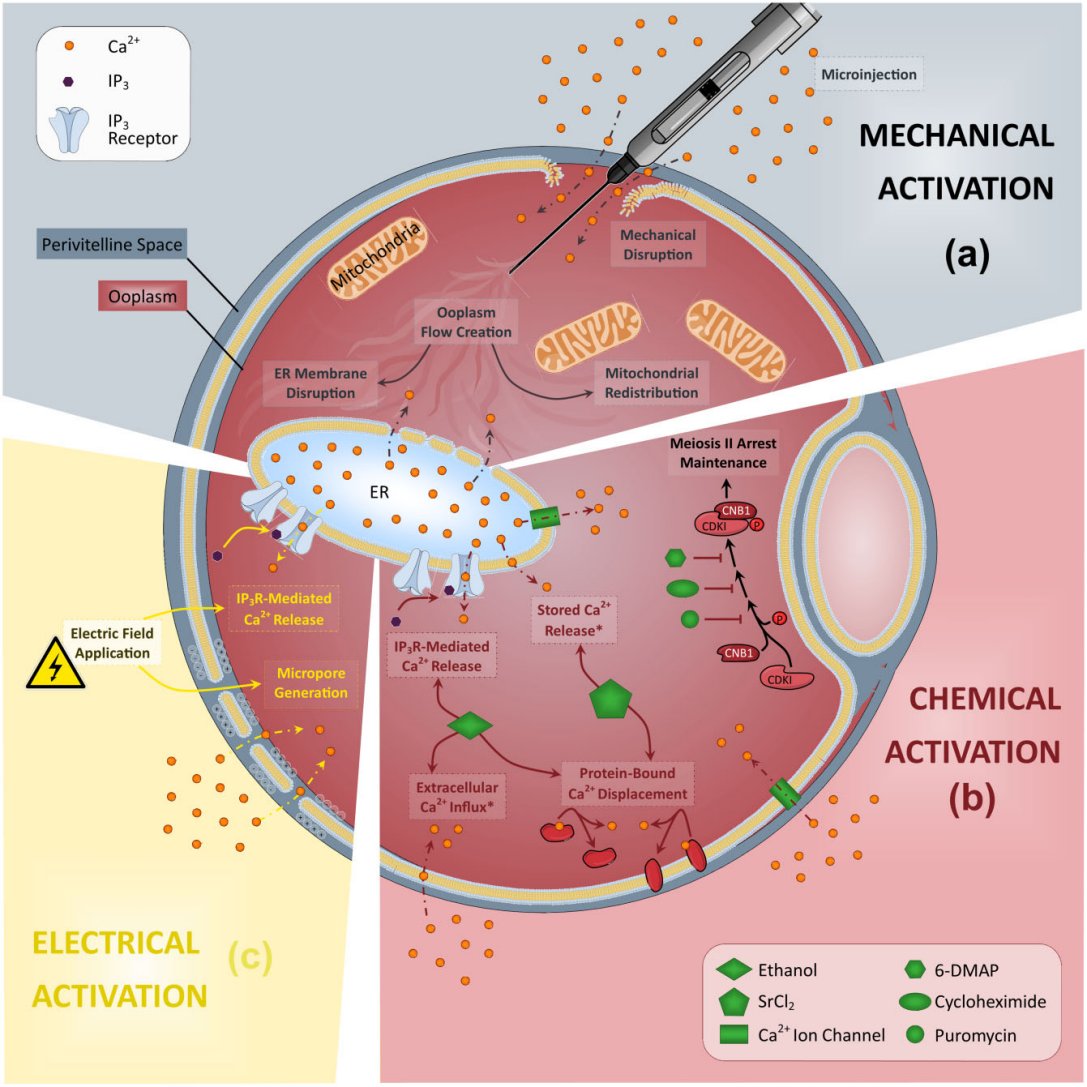
**Schematic representation of the purported mechanisms underlying the three most commonly applied methods of assisted oocyte activation.** (**a**) Mechanical activation usually involves a disruption of the plasma membrane and/or components within the oolemma, leading to an elevation of Ca^2+^ within the oocyte due to influx of Ca^2+^ and/or disruption of Ca^2+^ store membranes such as the endoplasmic reticulum (ER). **(b)** The mechanisms underlying chemical activation vary on the type of agent utilized, but usually involve the facilitated transport of extracellular Ca^2+^ into the oocyte either directly or via transport channels. **(c)** Electrical activation involves generation of pores within the oocyte membrane via application of varying electrical fields, allowing extracellular Ca^2+^ influx into the oolemma.

While the complete extent of the role of PLCζ in male fertility/infertility is currently the subject of much investigation (Kashir, 2020; Kashir *et al.*, 2020a; Meng *et al.*, 2020; Cheung *et al.*, 2020), it is clear that removal or abrogation of the pattern of Ca^2+^ release at oocyte activation underlies numerous cases of male infertility and abnormal embryogenesis (Kashir *et al.*, 2010). To this degree, AOA aims to trigger meiotic resumption in the oocyte by artificially elevating intracellular levels of Ca^2+^ (Ferrer-Buitrago *et al.*, 2018a,b). Mechanical, electrical, chemical or a combination of these stimuli each present different means of AOA (Fig. 3), and are associated with unique risks and benefits (Alberio *et al.*, 2000; Amdani *et al.*, 2013, 2016; Nasr-Esfahani *et al.*, 2010; Vanden Meerschaut *et al.*, 2014b).

Electrical activation usually involves the direct application of a voltage current, inducing charged lipid bilayer protein migration and pore formation in the membrane, enabling extracellular Ca^2+^ influx into the oolemma (Yanagida *et al.*, 1999, 2008; Egashira *et al.*, 2009; Vanden Meerschaut *et al.*, 2014b). Mechanical activation usually involves oolemma piercing using micromanipulation, followed by vigorous cytoplasmic aspiration using a modified ICSI procedure (Tesarik *et al.*, 2002; Ebner, 2004), eliciting a Ca^2+^ influx, usually followed by which ICSI is performed (Tesarik *et al.*, 1994; Neri *et al.*, 2014). Another mechanical method of activation is the microinjection of Ca^2+^ into the oocyte (Heindryckx *et al.*, 2008; Neri *et al.*, 2014). However, such methods are likely to be difficult to standardize, and as with most other physical methods, they will only induce a single Ca^2+^ increase (Kashir *et al.*, 2010). In contrast, chemical activation is mediated by Ca^2+^ ionophores, which are lipid-soluble molecules that can transport Ca^2+^ across the oolemma, by increasing Ca^2+^ permeability and causing extracellular Ca^2+^ influx, and elicit intracellular Ca^2+^ stores to release stored Ca^2+^ (Yoshida and Plant, 1992; Vanden Meerschaut *et al.*, 2013)_._

### Mechanical activation

Mechanical AOA involves modified ICSI techniques using a microinjection ICSI needle (Tesarik *et al.*, 2002), relying on oolemma piercing to elicit a calcium influx from the extracellular medium, following which ICSI is performed (Tesarik *et al.*, 1994; Nasr-Esfahani *et al.*, 2010; Neri *et al.*, 2014). A popular mechanical AOA method is to manually disrupt the plasma membrane, followed by vigorous cytoplasmic aspiration, increasing the oocyte Ca^2+^ load during injection and leading to higher fertilization rates (Tesarik *et al.*, 2002; Ebner, 2004). Such a methodology may also establish closer contact of the injected sperm with oocyte intracellular Ca^2+^ stores, enabling a more rapid diffusion of the physiological signalling pathway (Tesarik and Sousa, 1995). Less invasive techniques rely upon creating an ooplasm flow by mechanical disruption of Ca^2+^ stores (Tesarik *et al.*, 2002; Ebner, 2004). Another method for mechanical oocyte activation is the direct microinjection of Ca^2+^ into the oocyte (Heindryckx *et al.*, 2005, 2008; Neri *et al.*, 2014).

### Electrical activation

Electrical AOA protocols involve directly applied and/or alternate voltage currents to induce charged lipid bilayer proteins to move and form pores in the membrane, enabling extracellular Ca^2+^ flow into the oolemma (Yanagida *et al.*, 2008; Egashira *et al.*, 2009; Baltaci *et al.*, 2010; Vanden Meerschaut *et al.*, 2014b). A single electric pulse produces long-lasting, rapid Ca^2+^ elevations in the oocyte, which gradually return to baseline levels (Mansour *et al.*, 2009; Vanden Meerschaut *et al.*, 2014b), effectively parthenogenetically activating both human and mouse oocytes, inducing blastocyst formation (Versieren *et al.*, 2010) and significantly improving fertilization rates compared to standard ICSI (Baltaci *et al.*, 2010). Electrical resistance changes can also serve as a marker for confirming oocyte viability and penetration (Mor *et al.*, 2020). Technological advances in nanoscale electrostimulation have also allowed for a preferential targeting of intracellular membranes, without much effect on the plasma membrane (Batista Napotnik *et al.*, 2016), yielding high activation rates and improving parthenogenetic embryogenesis (Stein *et al.*, 2020).

However, such technologies in species other than mouse have yet to be tested (Stein *et al.*, 2020), while the overall efficiency of electrostimulation depends on multiple factors including pore size formed and ionic content of the surrounding medium. While such methods have successfully been applied on bovine and human oocytes (Yanagida *et al.*, 2008), these procedures may induce reactive oxygen species (ROS) within oocytes (Koo *et al.*, 2008). Furthermore, increases in Ca^2+^ are transient, with Ca^2+^ levels returning to basal values without the induction of oscillations (Neri *et al.*, 2014).

### Chemical activation

Chemical AOA protocols employ compounds that facilitate intracellular Ca^2+^ transients in the oolemma mediated via extracellular influx. Such agents are usually lipid-soluble compounds able to transport Ca^2+^ across cell membrane by increasing Ca^2+^ permeability and extracellular Ca^2+^ influx. Additionally, some compounds, such as IP_3_, also act on intracellular Ca^2+^ stores (Yoshida and Plant, 1992; Vanden Meerschaut *et al.*, 2013). Examples of popular chemical activating agents include ethanol, ionomycin and A23187 (by far the most popular clinical agents). Most agents result in a single prolonged Ca^2+^ rise but fail to elicit normal Ca^2+^ patterns. Other activating agents have been shown to cause multiple transients, and include strontium chloride (SrCl_2_) in mice (Kline and Kline, 1992b; Kishigami and Wakayama, 2007) and phorbol esters (Cuthbertson and Cobbold, 1985) or thimerosal (Fissore *et al.*, 1995). While strontium chloride’s mechanism of action in humans remains unclear (Swann, 2020), this compound is effective in mouse oocyte activation (Versieren *et al.*, 2010; Nikiforaki *et al.*, 2016; Kashir *et al.*, 2018).

Further compounds also used include 6-dimethylaminopurine (6-DMAP) and puromycin (Alberio *et al.*, 2000; Heindryckx *et al.*, 2008; Darwish and Magdi, 2015; Kim *et al.*, 2015; Yeste *et al.*, 2016a; Aydinuraz *et al.*, 2016; Capalbo *et al.*, 2016; Nikiforaki *et al.*, 2016; Yeste *et al.*, 2016b; Shang *et al.*, 2019), while exposure to low concentrations of ethanol also elicits a single rise in Ca^2+^, which also seems to increase the rate of high-quality embryo and blastocyst formation from fresh and vitrified human oocytes, *in*  *vitro* (Zhang *et al.*, 2017). The combination of multiple chemical mediators of AOA is able to produce embryos in animal models (Borges *et al.*, 2020a). Ca^2+^ ionophores, such as ionomycin or A23187, are the most commonly used form of AOA in IVF clinics today (Ebner *et al.*, 2012; D’Haeseleer *et al.*, 2014; Ebner *et al.*, 2015; Aydinuraz *et al.*, 2016; Capalbo *et al.*, 2016; Economou *et al.*, 2017; Karabulut *et al.*, 2018), and can work in conjunction with other compounds to enhance the processes important for fertility, such as acrosomal exocytosis (Akter *et al.*, 2019).

Thimerosal is another compound that induces Ca^2+^ release in several cell types and is capable of eliciting Ca^2+^ oscillations in mammalian oocytes (Swann, 1991) by increasing IP_3_R sensitivity to Ca^2+^ (Cheek *et al.*, 1993). However, thimerosal causes oxidation of tubulin, preventing polymerization and impairing spindle formation in oocytes (Alexandre *et al.*, 2003), and requires sequential treatment with dithiothreitol to prevent tubulin oxidation. While this does indeed successfully initiates activation that closely resembles fertilization signaling (McDougall *et al.*, 1993; Herbert *et al.*, 1997; Nakada and Mizuno, 1998; Stein *et al.*, 2020), the requirement for the use of a reducing reagent with thimerosal (which also prematurely terminates Ca^2+^ oscillations) has prevented its widespread clinical use (Stein *et al.*, 2020).

### Combined activation

Combinations of mechanical, electrical and/or chemical activation present innovative means of attempting to harness the unique benefits of each mode of activation, although with mixed results. One group (Heindryckx *et al.*, 2005) injected a small amount of CaCl_2_ with sperm during ICSI followed by exposure to ionomycin, leading to improved fertilization success rates. In another study, after AOA using electrical activation, ionomycin or SrCl_2_, the media was supplemented with cycloheximide and/or DMAP. However, while no differences were observed in activation and cleavage, higher morulation and blastulation rates were observed for both mouse and human oocytes (Versieren *et al.*, 2010). Another study utilized A23187 for AOA, supplementing the culture medium with granulocyte-macrophage colony stimulating factor (GM-CSF), increasing the number of high-quality embryos undergoing cleavage and blastulation. Analysis via array comparative genomic hybridization further suggested that exposure to GM-CSF after initial AOA could also result in fewer chromosomal abnormalities due to the cytogenic enhancing properties of GM-CSF (Economou *et al.*, 2017). A point to note is that the oocytes used in this study (upon exposure to AOA and GM-CSF) were relatively older (18–20 h post-oocyte retrieval). Although aged oocytes are an unavoidable reality for such studies (as failure to fertilize requires time to confirm), the age here is perhaps another factor to be mindful of.

## Clinical use of Ca^2+^ ionophores

By far, the most commonly used chemical means of AOA in both research and the clinic is A23187 (also known as calcimycin), a carboxylic antibiotic that binds divalent cations such as Ca^2+^ and Mg^2+^ and freely transports them across all biological membranes. The second-most common ionophore used in oocyte activation is ionomycin, which is far more specific for Ca^2+^ compared to A23187 and can activate and stimulate gene expression (Santella and Dale, 2015). A ready-to-use solution (CultActive), similar to A23187, has also been applied for clinical use with better success rates (Ebner *et al.*, 2012). However, ionomycin seems more potent and specific compared to A23187 (Kauffman *et al.*, 1980; Versieren *et al.*, 2010; Nikiforaki *et al.*, 2016).

Thus, while reports have been extensively described, the actual clinical applications of such chemicals remain limited. This is particularly problematic due to human oocytes not being particularly responsive to most of the aforementioned agents, relying on a combination of chemical treatments, coupled with sperm injection (Yamano *et al.*, 2000; Neri *et al.*, 2014). Furthermore, even following successful evocation of Ca^2+^ release in oocytes, most ionophores do not elicit the characteristic pattern of Ca^2+^ transients required for competent oocyte activation in humans, with only strontium chloride treatment in mice resulting in Ca^s+^ oscillations, accompanied by oocyte activation and efficient parthenogenesis (Bos-Mikich *et al.*, 1995; Ma *et al.*, 2005). The efficiency of strontium chloride in humans remains debatable, as no Ca^2+^ oscillations are observed (Rogers *et al.*, 2004). Strontium ions (Sr^2+^) are thought to gate oocyte IP_3_R1 receptors via the TRPV3 channel, which is thought to be involved in mediating Sr^2+^ influx in rodents (Brind *et al.*, 2000; Jellerette *et al.*, 2000; Carvacho *et al.*, 2013). However, considering that Sr^2+^ is thought to mediate oocyte activation via CaMKIIγ (Backs *et al.*, 2010), this very much remains a non-physiological mechanism of action.

Several studies have now examined the applicability of AOA within the clinic, for various chemical activating agents (Table I), with an equivalent body of research devoted to examining protocols using A23187 (Table II). One of the first reports on Ca^2+^ ionophore application examined ICSI couples characterized by poor fertilization rates, wherein ionophore treatment post-ICSI resulted in moderate zygote formation (Tesarik and Sousa, 1995). Subsequently, a study involving patients with a history of inconsistent fertilization and severe sperm morphological abnormalities used ionomycin to enhance fertilization but failed to generate good quality embryos (Moaz *et al.*, 2006). Conversely, however, separate studies examining cases of sperm defects and failed fertilization that were treated with CaCl_2_ injection concurrent with ICSI, followed by sequential Ca^2+^ ionophore treatments, showed increased fertilization rates and clinical pregnancies and births neonates (Heindryckx *et al.*, 2008; Nasr-Esfahani *et al.*, 2008; Mansour *et al.*, 2009; Neri *et al.*, 2014). Numerous case reports now exist demonstrating that ICSI combined with AOA greatly increases fertilization and subsequent pregnancy rates (Rybouchkin *et al.*, 1997; Kim *et al.*, 2001; Tesarik *et al.*, 2002; Heindryckx *et al.*, 2005; Kyono *et al.*, 2008; Tejera *et al.*, 2008; Taylor *et al.*, 2010), while a recent meta-analysis concluded that ionophore treatment significantly improved clinical pregnancy rates as well as oocyte activation (Murugesu *et al.*, 2017). However, this contradicted an earlier meta-analysis that suggested the opposite (Sfontouris *et al.*, 2015).

**Table I:** A comparative overview of study design and outcomes of AOA protocols using various chemical activators.

Endpoint type	Study type (AOA stimulus)	Fertilized oocytes (Total)	Experimental group (Total)	Control group	Primary findings	References
Efficacy	Retrospective (**Ionomycin**)	Undisclosed	History of severe teratozoospermia or previous ICSI failure (**50 couples**)	Standard ICSI	Rates of fertilization and transferable embryos increased with AOA Blastulation, pregnancy and implantation rates not improved.	Li *et al.* (2019b)
Safety	Retrospective (**Ionomycin**)	*In vitro* matured and transferred (**1228**)	Matched with controls for: - Female/male age - Female BMI - Duration of infertility number of transferred embryos (total and per cycle) - Type of embryo transferred (cleavage embryo and blastocyst), - Endometrial thickness on embryo transfer day - Type of endometrial preparation - Causes of infertility (**676 women**)	Standard ICSI	Rates of: - Biochemical pregnancy - Clinical pregnancy, - Implantation - Miscarriage, - Ectopic pregnancy - Multiple pregnancy - live births Not significantly increased	Li *et al.* (2019a)
Safety	Retrospective (**Ionomycin**)	*In vivo* matured (**Undisclosed**)	History of teratozoospermia, severe male fertility or sperm obtained via testicular sperm extraction (**1681 couples**)	Standard ICSI	Rates of: - Abortion, - Major birth defects - Developmental retardation Not significantly increased	Deemeh *et al.* (2015)
Efficacy	Prospective, Multi-Centre (**Ionomycin**)	*In vivo* matured (**193**)	History of ICSI or low fertilization rate. (**14 couples**)	Standard ICSI—split by sibling oocytes	Fertilization rates in patients with low fertilization history not always increased, even upon pre-screening for OAD.	Vanden Meerschaut *et al.* (2012)
Safety	Retrospective (**Ionomycin with CaCl_2_ injection during ICSI**)	*In vivo* matured (**undisclosed**)	No fertility history (**undisclosed**)	Natural conception	No intellectual or language disabilities identified in AOA children	D’Haeseleer *et al.* (2014)
Safety	Retrospective (**Ionomycin with CaCl_2_ injection during ICSI**)	*In vivo* matured (**Undisclosed**)	History of total ICSI failure, near total ICSI failure, or globozoospermia (**14 couples**)	Natural conception	Cognitive, language, motor development and behaviour within general population standards	Vanden Meerschaut *et al.* (2014)
Safety	Prospective (**A23187 vs. Ionomycin**)	*In vitro* matured (**231**)	History of poor sperm quality, female factor infertility, or idiopathic infertility (**35 women**)	None	Congenital malformations detected in 6.3% of children born following ionomycin treatment.	Mateizel *et al.* (2018)
Safety and Efficacy	Prospective (**Ionomycin with CaCl_2_ injection during ICSI**)	*In vivo* matured (**1110**)	History of ICSI or low fertilization rate due to poor sperm quality. (**30 women**)	Standard ICSI from previous cycles	Fertilization and pregnancy rates back to normal. No detectable minor or major congenital defects in babies.	Heindryckx *et al.* (2008)
Efficacy	Prospective (**A23187 with GM-CSF vs. Ionomycin**)	*In vitro* matured from humans and in vivo matured from mice (**69 human, 40 mouse, 420 mouse parthenotes**)	No history of ICSI failure (**42 women**)	ICSI with activation- capable (control) or activation-deficient sperm	Mice and human oocytes responded differently to the two ionophores. Mouse oocyte activation and blastulation higher using ionomycin compared to A23187. Neither ionophore restored normal fertilization rates in human *in vitro* matured oocytes.	Nikiforaki *et al.*, (2016)
Safety and Efficacy	Prospective (**A23187 vs. SrCl_2_**)	*In vivo* matured (**931**)	History of ICSI failure or low fertilization rate (**50 women for calcimycin, 35 women on SrCl_2_, 530 women on ICSI without AOA**)	Standard ICSI	Both improved fertilization rates Neither increased - Pregnancy - Implantation - Miscarriage Children had no congenital/cognitive abnormalities compared to controls.	Kyono *et al.* (2012)
Efficacy	Prospective (**Ionomycin vs. Ionomycin with SrCl_2_**)	*In vitro* matured (**1170**)	History of infertility with 99%-100% abnormal sperm morphology (**66 on ionomycin, 39 on ionomycin with SrCl_2_**)	Standard AOA with Ionomycin (**no SrCl_2_**)	Ionomycin alone gave higher rates of oocyte activation compared to Ionomycin and SrCl_2_ Treatment with SrCl_2_ improved embryo quality rather than with just Ionomycin.	Norozi-Hafshejani *et al.* (2018)
Efficacy	Prospective (**7% ethanol**)	*In vitro* matured, fresh, or vitrified (**810**)	No fertility history (**325 women**)	Standard ICSI	No improvement in fertilization or cleavage rates Increased rates of high-quality embryogenesis (from both fresh and vitrified oocytes).	Zhang *et al.* (2017)
Efficacy	Prospective (**7% ethanol**)	Originally failed to mature, were vitrified, and then matured *in-vitro* (**386**)	No fertility history (**undisclosed**)	Standard ICSI	Significantly improved high-quality embryo and blastocyst formation rates from vitrified oocytes to those comparable to fresh oocytes.	Liu *et al.* (2013)

AOA, artificial oocyte activation; GM-CSF, granulocyte-macrophage colony stimulating factor; OAD, oocyte activation deficiency.

**Table II:** An overview of study design and outcomes of AOA protocols utilizing A23187, CultActive and protocols supplemented with GM-CSF.

Endpoint type	Study type	Fertilized oocytes (Total)	Experimental group (Population)	Control group	Primary findings	References
Efficacy	Prospective (**A23187**)	*In vitro* matured (**333**)	No fertility history (**26 women**)	Standard ICSI	Improved fertilization rate. Cleavage score and embryo quality remained unchanged.	Nazarian *et al.* (2019)
Efficacy	Prospective (**A23187**)	*In vitro* matured (**40**)	History of globozoospermia (**5 women**)	None	Treatment successfully produced live births for males with globozoospermia.	Shang *et al.* (2018)
Safety	Retrospective (**A23187**)	*In vivo* matured (**Undisclosed**)	History of ICSI failure or low fertilization rate without oocyte abnormality (**678 pregnancies**)	Standard ICSI	No increase in: - Foetal defects including structural or chromosomal malformations) - Unhealthy newborns - First and second trimester abortions - Intrauterine foetal death - Ectopic/chemical pregnancies - Gestational age - Birth weight - Newborn gender	Miller *et al.* (2016)
Safety	Prospective (**A23187**)	*In vitro* matured, vitrified (**49**)	Endometriosis, male factor, tubal, or idiopathic aetiologies of infertility (**12 women**)	IVF	No increase in number of chromosome segregation errors in meiosis II. Evidence suggested affected second polar body extrusion.	Capalbo *et al.* (2016)
Safety and Efficacy	Retrospective, Case-control (**A23187**)	*In vivo* matured (**>180**)	History of ICSI failure or low fertilization rate (**45 women**)	Standard ICSI from previous cycles	Improved fertilization rates No change in: - Cleavage rates - Number of live births. - Abortion rates - Congenital anomalies	Sdrigotti *et al.* (2015)
Safety	Retrospective (**A23187**)	*In vivo* matured or unfertilized, aged oocytes post-ICSI (**79**)	History of total or near total ICSI failure (**3 women**)	None	All AOA-born children presented normal: - Physical and mental development - Normal chromosome ploidy No increase in genetic variations or chromosomal alterations	Lu *et al.* (2014)
Safety and Efficacy	Unblinded Clinical Trial (**A23187**)	*In vivo* matured (**313**)	Teratozoospermia (**31 women**)	Standard ICSI	No change in rates of: - Implantation - Fertilization - Pregnancy rates - Multiple pregnancies - Spontaneous abortion	Eftekhar *et al.* (2013)
Safety and Efficacy	Retrospective (**A23187**)	*In vivo* matured (**2360**)	History of ICSI failure or low fertilization rate (**185 women**)	Standard ICSI from previous cycles	Improved fertilization and implantation rates No congenital birth defects observed.	Yoon *et al.* (2013)
Safety and Efficacy	Retrospective (**A23187**)	*In vivo* matured (**1476**)	History of ICSI failure or low fertilization rate. (**89 women**)	Standard ICSI from previous cycles	Improved rates of: - Fertilization, - Implantation and pregnancy No change in: - Abortion rates - Birth weight - Malformation rates	Montag *et al.* (2012)
Safety and Efficacy	Prospective (**A23187 with GM-CSF**)	Aged, unfertilized oocytes post-ICSI (**18 assessing safety,** **140 assessing efficacy**)	No fertility history (**66 couples**)	Standard ICSI	Standard AOA resulted in chromosomal abnormalities in all embryos Supplementation with GM-CSF improved rates of: - Activation - Cleavage - High quality embryos - Embryo development - Blastulation 62.5% GM-CSF supplemented embryos were chromosomally normal.	Economou *et al.* (2017)
Safety and Efficacy	Prospective, Retrospective, Multi-Center (**CultActive**)	*In vitro* matured (**138**)	Low oocyte count, OAT, or frozen sperm (**1837 women**)	Standard ICSI	Improved fertilization and pregnancy rates, and comparable embryogenesis.	Karabulut *et al.* (2018)
Safety and Efficacy	Prospective, Single Blind (**CultActive**)	*In vivo* matured, then fertilized by IMSI (**49**)	History of low fertilization and teratozoospermia (**12 women**)	Standard ICSI and split by sibling oocytes	No improvement in fertilization rates and impeded embryogenesis quality.	Aydinuraz *et al.* (2016)
Efficacy	Prospective (**CultActive**)	*In vivo* matured (**77**)	History of ICSI failure. Fresh or frozen sperm obtained via testicular sperm extraction (**4 women**)	Standard ICSI from previous cycles	Successful: - Pronuclei production and fusion - Cleaved blastomeric stage transition - Progressive embryogenesis	Darwish and Magdi (2015)
Safety and Efficacy	Prospective, Multi-Center (**CultActive**)	*In vivo* matured (**2071**)	History of ICSI failure (**101 women**)	Standard ICSI from previous cycles	Improved rates of: - Fertilization - Implantation - Pregnancy No change in: - Embryo quality - Rates of malformation.	Ebner *et al.* (2015)
Safety and Efficacy	Prospective, Multi-Center (**CultActive**)	*In vivo* matured (**1370**)	History of ICSI failure or low fertilization rate (**66 women**)	Standard ICSI from previous cycles	Improved rates of: - Fertilization - Implantation - Pregnancy No change in: - Embryo quality - Rates of malformation.	Ebner *et al.* (2012)

AOA, artificial oocyte activation; GM-CSF, granulocyte-macrophage colony stimulating factor; IMSI, intra-cytoplasmic morphologically selected sperm injections; OAT, oligoasthenoteratozoospermia.

There seems to be an overall lack of consensus regarding the efficacy of improvements in fertilization and pregnancy rates following AOA (Neri *et al.*, 2014). Furthermore, Vanden Meerschaut *et al.* (2012) indicated that AOA may not benefit all patients experiencing OAD, with fertilization history and sperm parameters seemingly playing an important role (Vanden Meerschaut *et al.*, 2012, 2013; Neri *et al.*, 2014). Thus, it is not yet clear which group of AOA patients would be most likely to benefit, apart from severe cases of OAD, without further clinical investigations. Indeed, current opinion with regards to this is split within the literature. A recent prospective multi-centre study concluded that Ca^2+^ ionophore treatment successfully increases clinical pregnancy and live-birth rates in patients with low or failed fertilization (Ebner *et al.*, 2015).

Fertilization and pregnancy rates following AOA seem highly variable, most likely due to the heterogenic and low number of patients recruited in the vast majority of studies, with differences between patient baseline characteristics and activating agents employed, making it hard to compare different reports (Vanden Meerschaut *et al.*, 2013). AOA protocols used throughout the published literature diverge in the ionophore concentration used, duration of ionophore exposure, the moment of ionophore exposure following ICSI and the number of ionophore exposures (Vanden Meerschaut *et al.*, 2013). Thus, it appears likely that while AOA can be significantly effective to resolve at least cases of extreme OAD, further detailed and focused investigations are required to ascertain specific protocols for all groups of patients. Perhaps ionophore treatment success is related to fertilization rates in previous cycles, with AOA presenting with the best results in patients with a history of < 30% fertilization in a previous ICSI cycle (Vanden Meerschaut *et al.*, 2012; Ebner *et al.*, 2015).

## Efficacy and safety of AOA

**Figure 4.**
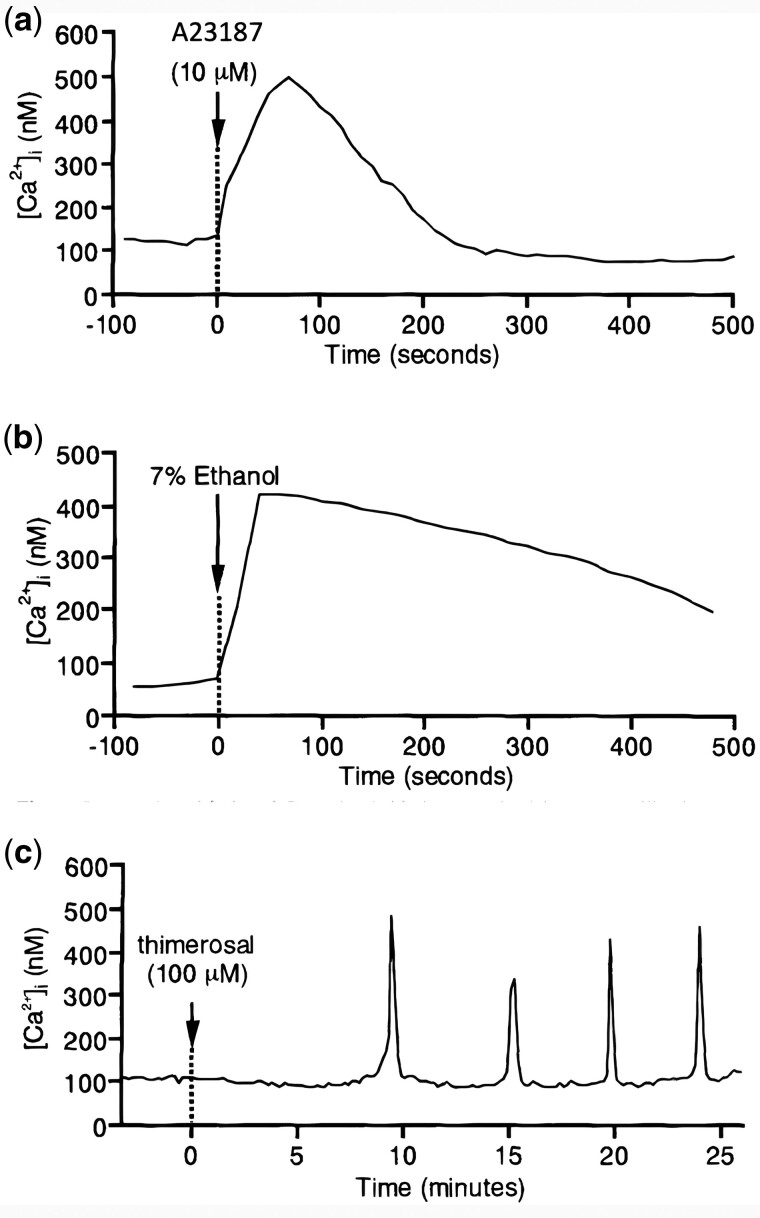
**Representative Ca^2+^ responses in mammalian oocytes following treatment**. Treatment with **(a)** A23187, **(b)** 7% ethanol and **(c)** thimerosal. Figure reproduced from Nakada and Mizuno (1998) with permission.

An obstacle to the widespread use of AOA is that the safety and efficacy of such practice is not yet fully established, with a dearth of randomized control trials and follow-up studies leaving the safety and efficacy of AOA unclear (Vanden Meerschaut *et al.*, 2012; Santella and Dale, 2015; Sfontouris *et al.*, 2015; van Blerkom *et al.*, 2015; Aydinuraz *et al.*, 2016; Ebner and Montag, 2016; Ferrer-Buitrago *et al.*, 2018a,b). A further concern is that the Ca^2+^ oscillation pattern produced by AOA is distinct from that of physiological oocyte activation, whereby most Ca^2+^ ionophores release Ca^2+^ from intracellular stores in a temporally and spatially uncontrolled fashion in a single ‘tidal wave-like’ flow that does not correspond to the physiological activation process (Fig. 4) (Versieren *et al.*, 2010; Ebner *et al.*, 2012; Santella and Dale, 2015; van Blerkom *et al.*, 2015). Perhaps the most reason to be cautious is that most ionophores are toxic to oocytes followed prolonged exposure (Steinhardt *et al.*, 1974; Swann, 2018), and thus care must be taken to ensure the correct dosages and timings are applied.

Given the complexity of cellular mechanisms regulated by intra-cytoplasmic Ca^2+^ levels and oscillations (Alberio *et al.*, 2000), Ca^2+^ ionophores have raised concerns of molecular downstream consequences, issues with long-term gene expression and epigenetic alterations (i.e. DNA methylation), with possible implications affecting embryonic development post-implantation (Ebner *et al.*, 2012; Vanden Meerschaut *et al.*, 2014b; Santella and Dale, 2015; Capalbo *et al.*, 2016; Nikiforaki *et al.*, 2016; Anifandis *et al.*, 2019). For example, protein synthesis and degradation changes in the first cell cycle may occur as a result of AOA, based on data with parthenogenetically activated mammalian oocytes (Ebner and Montag, 2016). Furthermore, the effects of ionophores upon oocyte mitochondrial metabolism and cellular homeostasis remain unknown (Santella and Dale, 2015; van Blerkom *et al.*, 2015; Kashir *et al.*, 2018).

Mammalian oocyte activation involves a concerted profile of Ca^2+^ oscillations, with characteristic frequencies and amplitude of each transient (Kashir *et al.*, 2013a, 2014), released in an IP_3_-dependant manner. Several animal studies have demonstrated that the number and amplitude of Ca^2+^ transients not only affects activation efficiency, but also profoundly influences subsequent embryonic development (Ducibella *et al.*, 2002; Ozil *et al.*, 2006; Kim *et al.*, 2011b), blastocyst quality (Bos-Mikich *et al.*, 1997) and the implantation potential of rabbit parthenogenotes (Ozil and Huneau, 2001) and mouse zygotes, resulting in altered embryonic gene expression (Ozil *et al.*, 2006). Indeed, a higher activation rate does not necessarily correlate to a higher birth rate, which is potentially regulated by mechanisms within the oocyte activation process that affects later developmental stages (Yamamoto *et al.*, 2020).

Although embryo quality does not seem to differ between the use of AOA or standard ICSI (Ebner *et al.*, 2012, 2015; Ebner and Montag, 2016; Karabulut *et al.*, 2018), there were only limited advantages for the application of AOA regarding fertilization rates (Karabulut *et al.*, 2018). For example, although a recent study comparing the spindle-chromosome normalcy and IP3R1 distribution among embryos that failed either traditional ICSI or ICSI followed by AOA was unable to conclude a significant difference in Ca^2+^ releasing deficiencies between both groups, it identified a downstream activation deficiency that could not be overcome by Ca^2+^ ionophores alone (Ferrer-Buitrago *et al.*, 2019). Both the efficacy and safety of AOA procedures have come under scrutiny, warranting further studies on the reproductive technique before widespread application in patients (Vanden Meerschaut *et al.*, 2012; Santella and Dale, 2015; Sfontouris *et al.*, 2015; van Blerkom *et al.*, 2015; Aydinuraz *et al.*, 2016; Capalbo *et al.*, 2016; Ferrer-Buitrago *et al.*, 2018b).

Recent studies have also indicated, at least on the face of it, that utilizing AOA does not significantly alter the morphokinetic parameters of embryos resulting from either ionomycin (Martínez *et al.*, 2021) or calcimycin (A23187) (Shebl *et al.*, 2021), with AOA embryos developing normally for most milestones. However, a key difference when utilizing ionomycin was that the time taken for second polar body extrusion (tPB2) and the third cell cycle (t3) were both significantly faster compared to the normal ICSI groups (Martínez *et al.*, 2021), which perhaps is a reflection of the rapid and non-physiologic release of Ca^2+^ associated with use of ionophores (Kashir *et al.*, 2020b; Martínez *et al.*, 2021). Similarly, utilization of ready-to-use A23187 (calcimycin) solution did not result in major differences between groups, with most morphokinetic parameters exhibiting convergence, with the exception of the time taken for pronuclear formation (which was faster in the ionophore group) and third cell cycle (s3 in this study) (Shebl *et al.*, 2021). It should be noted that pregnancy rates between ionophore and control groups remained non-significant, requiring more studies before such morphokinetics can be linked to pregnancy success.

The use of A23187 for AOA has raised mixed concerns over safety and the need for varying degrees of risk management. A23187 exposure has been shown to degrade embryos, and could underlie the risk of failed second polar body extrusion by lack of coordination with telophase in MII, although there has been no increase in chromosome segregation errors (Aydinuraz *et al.*, 2016; Capalbo *et al.*, 2016). When A23187 use was supplemented with GM-CSF, 62.5% of embryos were free of chromosomal abnormalities (Economou *et al.*, 2017). Other studies have failed to link chromosomal malformations with AOA and A23187, by focusing on later stages of development, gestational outcomes, neonatal health (Miller *et al.*, 2016), rates of medical abortions, birth defects, congenital malformations and multiple pregnancy risk, all indicating no significant change between cases without AOA (Sato *et al.*, 2011; Ebner *et al.*, 2012; Yoon *et al.*, 2013; Deemeh *et al.*, 2015; Ebner *et al.*, 2015; Matsukawa *et al.*, 2015; Sdrigotti *et al.*, 2015; Miller *et al.*, 2016).

Chromosomal alterations in children born from AOA were similar to those of their parents (Lu *et al.*, 2014), while such children exhibited normal physical and mental development, as well as comparable language, cognitive and behavioural abilities to the general population (D’Haeseleer *et al.*, 2014; Vanden Meerschaut *et al.*, 2014b). In a decade-long study of children born from either A23187 or ionomycin use, only 6.3% of children had congenital malformations, all from treatment of ionomycin (Mateizel *et al.*, 2018). Mouse zygotes treated with ionomycin exhibited normal embryogenesis and development of fertile pups (Heytens *et al.*, 2008). However, application of high concentrations of ionomycin following mouse sperm ICSI increased the frequency and amplitude of Ca^2+^ release, influencing mitochondrial metabolism, increasing reactive oxygen species (ROS) and decreasing ATP, and impairing blastocyst formation (Chen *et al.*, 2020).

Other reports showed no adverse effects following the application of ionomycin, electrical pulses or strontium chloride in an activation-deficient mouse model, resulting in normal development and fertile pups (Vanden Meerschaut *et al.*, 2013). However, ionomycin-induced Ca^2+^ transients in starfish eggs were followed by rapid alterations of the actin cytoskeleton, while cortical granules were disrupted or fused with other vesicles. This collectively prevented egg cortical maturation, despite normal fertilization progression, with monospermic zygotes failing to proceed past the first cell division or displaying problematic subsequent cell cleavage (Vasilev *et al.*, 2012; Santella and Dale, 2015). Considering that actin cytoskeletal dynamics have been linked intricately with profiles of Ca^2+^ oscillations in mammalian fertilization (Ajduk *et al.*, 2011; Swann *et al.*, 2012), similar investigations are required in the mammalian scenario to ensure embryogenic competency is maintained following use of similar protocols in humans.

Examining the safety and efficacy of ionomycin, ethanol, electrical activation and combinations of activators reveals the experimental nature of AOA procedures. Notably, oocyte Ca^2+^ analysis may be a valid tool, together with the heterologous mouse oocyte activation test (MOAT), to pre-screen patients with ICSI fertilization failure before proceeding to AOA (Ferrer-Buitrago *et al.*, 2018a,b). Most ionophores are capable of uncontrollably releasing Ca^2+^ from all intracellular stores, including those not normally involved in activation, potentially affecting factors downstream of the spatial and temporal regulation of Ca^2+^ transients (Ducibella and Fissore, 2008; Santella and Dale, 2015). Finally, ionophores exert multiple effects on cellular homeostasis, the effects of which require investigation in oocytes, which may exert long-term genetic/epigenetic, biochemical and physiological effects (Santella and Dale, 2015). It is thus essential that such downstream effects upon progeny are examined, which is admittedly difficult to do for numerous reasons, the major one being that AOA protocols are a relatively recent development in ART.

While some limited population studies have been performed on humans, the sizes of populations were quite small, ranging from 10 to 25 children being examined. Furthermore, such studies examine gross developmental and behavioural parameters, with potential differences in gene expression, epigenetic modifications and molecular alterations still requiring investigation. One group (Isom *et al.*, 2013) examined the transcriptional profile of porcine embryos generated via a series of ART methodologies, finding significant alterations compared to normal embryos, including downregulation of the transforming growth factor β signalling in IVF embryos, including aberrant regulation of ubiquitin-mediated proteolysis and ErbB signalling. Significantly, however, expression of genes involved in chromatin modification, RNA-mediated gene silencing and apoptosis were significantly disrupted in embryos created via somatic cell nuclear transfer, which involved the use of AOA. Until further assessment of the efficacy and safety of AOA is conducted, AOA will remain limited and confined to select clinical applications.

## Assessment of AOA efficacy and safety

Although numerous studies have attempted to assess the safety and efficacy of AOA, major challenges must be overcome before broad application within clinics. These include heterogeneity in varied experimental methodologies and inconsistent patient inclusion criteria between research groups, as well as nonstandard endpoint choices in assessing safety outcomes. Combined with the small sample size of many studies and the limited applicability of animal models for explaining biological processes in humans, such challenges must be understood and addressed before the efficacy and safety of AOA can be improved.

### Heterogeneity in study methodology

The lack of homogeneity in AOA study design is evident in the range of protocol variants in AOA studies, even when subdivided by activation type. No industry standard is present for study methodology, further compounding this issue. This stems from a lack of homogeneity in protocols for other aspects of ART. In the case of chemical activation, there is a significant variety among chemicals used, chemical concentration, timing and duration of exposure, and number of times the chemical is applied (Heindryckx *et al.*, 2008; Liu *et al.*, 2011; Ebner *et al.*, 2012, 2015; Kyono *et al.*, 2012; Montag *et al.*, 2012; Vanden Meerschaut *et al.*, 2012; Yoon *et al.*, 2013; Darwish and Magdi, 2015; Aydinuraz *et al.*, 2016; Capalbo *et al.*, 2016; Miller *et al.*, 2016; Nikiforaki *et al.*, 2016; Economou *et al.*, 2017; Zhang *et al.*, 2017; Karabulut *et al.*, 2018).

Such variation not only exists between studies but also within the same study among different patients recruited. In addition, the choice of using either laboratory-generated chemical activators or commercially available options, such as CultActive (whose exact concentration remains undisclosed by the manufacturer, although Nikiforaki *et al.* (2016) estimated this to be at least >15 μmol/l), is another source of variability. The rise in amplitude of Ca^2+^ is more standardized and reproducible under laboratory conditions, but commercial chemical activators would be used in the clinical setting and may produce differing results (Ebner *et al.*, 2012; Aydinuraz *et al.*, 2016; Goksan Pabuccu *et al.*, 2016; Nikiforaki *et al.*, 2016).

Electrical AOA is also constrained by non-standardized activation methodologies, although the techniques themselves differ from those of chemical activation. Variance in the types of electrical pulses, time duration of pulses and repetition rates of pulses all contribute to the heterogeneity of studies (Mansour *et al.*, 2009; Baltaci *et al.*, 2010; Versieren *et al.*, 2010). Additionally, although studies that combine different activation techniques present novel means of overcoming OAD, it becomes increasingly difficult to compare the safety and efficacy of these studies with others that rely on only one activation mechanism for AOA. Studies experimenting with various combinations of chemical and mechanical activation, administration of different chemical activators and different orders of administration of chemical activators all suffer from a lack of standardized means for evaluating safety (Tesarik *et al.*, 2002; Kyono *et al.*, 2008). Nonetheless, the importance of investigating combinations of stimuli for AOA necessitates such studies.

### Inconsistent sample inclusion criteria

Diverse inclusion criteria for patient samples across studies further complicate matters, particularly as gametes used in ICSI and AOA are subject to a variety of treatments and procedures. There are a multitude of mechanisms for sperm selection without homogeneity in methodology (Oseguera-López *et al.*, 2019; Vaughan and Sakkas, 2019). For example, immature testicular sperm require exposure to higher levels of the activating compound than ejaculated or epididymal sperm, as well as lower sperm retrieval rates, suggesting the need for careful protocol adjustment (Boeri *et al.*, 2020). Immature oocyte incidence may also pose a similar issue (Braga *et al.*, 2020). Furthermore, testicular spermatozoa are expected to be in better condition as they have not yet been exposed to post-testicular DNA fragmentation (Agarwal *et al.*, 2020). Thus, this variation in sperm quality between samples makes it difficult to have a standard comparison for verifying safety and efficacy across studies.

Female gametes also suffer from such variation among study samples, although standard protocols require use of freshly collected oocytes arrested at MII. However, the impact of AOA on oocytes matured *in*  *vitro* (IVM) after vitrification and cells cryopreserved with ethylene glycol and sucrose or vitrified with sucrose or trehalose as cryoprotectants have also been studied (Liu *et al.*, 2011; Capalbo *et al.*, 2016; Zhang *et al.*, 2017). Moreover, a dearth of gametes for experimentation has led to the use of IVM oocytes derived from stimulated cycles or aged oocytes that remain unfertilized post-ICSI for experiments; these samples are used to evaluate chemical activator efficacy or AOA safety and efficiency (Nikiforaki *et al.*, 2016; Economou *et al.*, 2017).

Additionally, gametes are routinely pre-screened prior to ICSI to improve outcomes, adding bias to cross-study evaluations of AOA efficacy. For instance, intra-cytoplasmic morphologically selected sperm injections can select sperm based on fine morphological features under high magnification and the activation capacity of sperm can be selected for via MOAT (Heindryckx *et al.*, 2008; Versieren *et al.*, 2010; Vanden Meerschaut *et al.*, 2012; Aydinuraz *et al.*, 2016). This is further complicated by the fact that sperm with specific morphologies tend to exhibit different capacities for oocyte activation, even within the same sample (Kashir *et al.*, 2010, 2012; Kashir, 2020; Sermondade *et al.*, 2011; Vanden Meerschaut *et al.*, 2013). When such techniques are employed as a screen for patient samples before the start of a study, it is improper to compare these results to those of other studies that do not have any such screen in place.

Varying grades of male factor infertility are accepted as inclusion criteria, with most studies not further subdividing patient groups according to infertility aetiology and/or fertilization failure rate. Each patient’s history includes information on the incidence of teratozoospermia, asthenozoospermia, oligozoospermia, cryptozoosperia, azoospermia, globozoospermia, female factor infertility and/or any other unknown factor infertility (Esteves *et al.*, 2018; Esteves and Roque, 2019; Lin *et al.*, 2019; Moretti *et al.*, 2019; Stimpfel *et al.*, 2019; Fesahat *et al.*, 2020; Morin *et al.*, 2020). Many studies include all couples with a history of ICSI fertilization failure, regardless of aetiology, and some do not even specify the specific inclusion criteria besides a general history of ICSI failure. In fact, a few studies have recommended AOA treatment for patients even when the couple has no history of previous ICSI failure (Eftekhar *et al.*, 2013; Karabulut *et al.*, 2018).

Finally, inconsistencies in patient group inclusion can arise due to different criteria for control groups between studies. Often, due to a dearth of embryos and the ethical considerations of producing many embryos for research, data from previously failed ICSI cycles are employed as controls in many efficacy and safety studies. Alternatively, some studies use sibling oocyte split randomizations, in which oocytes from the same woman are divided and assorted into experimental or control groups, with the latter undergoing standard ICSI. Standard ICSI performed on different patients and the babies born from this procedure are used as controls for both prospective and retrospective studies assessing safety. Assessing the long-term safety of AOA using children born through standard ICSI or children from the general population as controls can vary significantly in terms of the baseline outcome for comparison, introducing yet another source of variability.

### Non-standardized outcome assessments

While efficacy endpoints classically include fertilization, cleavage, embryo transfer, implantation and clinical pregnancy rates, as well as embryo and blastocyst quality assessments, the criteria for evaluating safety outcomes are much more variable. Commonly cited outcome measures for safety include miscarriage rates, congenital and neonatal malformation occurrence, as well as chromosomal analysis and cytogenetic analysis, although endpoint reporting is not carried out in a consistent manner. As for the long-term follow-up of children born through AOA, studies are scarce; still, those conducted till date include a variety of analyses, such as physical, mental, cognitive, language, behavioural and motor development. Variations in AOA methods and patient inclusion criteria prohibit the pooling of results from different studies. In a study using couples with a history of TFF, only 10 of 690 electronically identified records were eligible for direct comparison between ICSI and ICSI-AOA. Hence, no robust conclusions could be drawn for both AOA efficacy and safety (Sfontouris *et al.*, 2015). This undoubtedly constitutes one of the largest obstacles to the establishment of a standard clinical protocol for measuring the safety of AOA.

### Restricted sample size

As AOA is only recommended for only select patients, this severely restricts the availability of embryos for efficacy and safety assessments. Thus, although a large number of studies have been conducted, each has a small sample size (Versieren *et al.*, 2010; Ebner *et al.*, 2012; Kyono *et al.*, 2012; Eftekhar *et al.*, 2013; Vanden Meerschaut *et al.*, 2014a,b; Darwish and Magdi, 2015; Aydinuraz *et al.*, 2016; Economou *et al.*, 2017; Karabulut *et al.*, 2018). In conjunction with ethical restrictions on mass producing embryos for research, the small sample size of studies explains the difficulty establishing optimal control groups with randomization (Sfontouris *et al.*, 2015). After all, embryos fertilized via standard ICSI are not abundant nor readily available for research purposes. It follows that the clinical follow-up of children born from AOA is even more limited than analyses of embryo or neonatal safety. The rarity of children born via AOA compared to the general population renders large-scale studies comprehensibly scarce, complicates the establishment of sound controls and limits the ability to draw conclusions of statistical significance across studies (Heindryckx *et al.*, 2008; Yoon *et al.*, 2013; D’Haeseleer *et al.*, 2014; Vanden Meerschaut *et al.*, 2014a,b; Deemeh *et al.*, 2015; Sfontouris *et al.*, 2015; Capalbo *et al.*, 2016).

### Animal model limitations

Animal models are a routinely used alternative to the use of human gametes for AOA studies (Tesarik *et al.*, 2002; Versieren *et al.*, 2010; van Blerkom *et al.*, 2015; Capalbo *et al.*, 2016; Nikiforaki *et al.*, 2016; Ogura, 2020). However, numerous differences exist between oocyte activation mechanisms of humans and commonly employed animal models, such as mice (Vanden Meerschaut *et al.*, 2013, 2014b; van Blerkom *et al.*, 2015; Nikiforaki *et al.*, 2016; Kashir *et al.*, 2018). Above a certain Ca^2+^ threshold, mouse and rabbit oocyte activation efficiency are independent of amplitude, pattern and the duration of a rise in Ca^2+^ levels, although embryo/blastocyst quality, long-term gene expression and development to term are affected (Heindryckx *et al.*, 2008; Nikiforaki *et al.*, 2014, 2016). Such variation among species suggests the need for the use of utmost caution in extrapolating results to the human condition from animal models. Indeed some ionophores, such as strontium chloride (Sr^2+^), may be effective in mouse models, but may not yield consistent/successful results in humans (Lu *et al.*, 2018b).

## Alternative AOA avenues

Apprehension towards AOA protocols and the need for more robust and reliable studies to support the technique are consequences of the artificial mechanism of action of AOA, which creates non-physiological patterns of Ca^2+^ release. To this degree, it is of utmost importance that studies focus not only on further elucidating the mechanistic modalities underlying Ca^2+^ release and regulation, but also establish more physiologically relevant modalities of eliciting the required pattern of Ca^2+^ release at fertilization.

### PLCζ-based AOA strategies

A goal of ART is to mimic as closely as possible the physiological processes that occur throughout mammalian fertilization, making PLCζ a potential replacement for most current agents of AOA as a physiologically relevant mechanism within the clinic. Several reports have linked defects in human PLCζ with cases of OAD. Indeed, sperm from men who routinely fail IVF and/or ICSI either fail to elicit Ca^2+^ oscillations in oocytes or do so in an uncharacteristic or abnormal profile (Yoon *et al.*, 2008; Heytens *et al.*, 2009). Importantly, sperm from such patients exhibit reduced or absent levels of PLCζ within the sperm head (Yoon *et al.*, 2008; Heytens *et al.*, 2009; Kashir *et al.*, 2011), while mutations in PLCζ may be contributing not only to male infertility but also perhaps to cases of male sub-fertility (for a detailed review, see Kashir (2020)). Furthermore, levels of PLCζ in sperm and the proportion of sperm exhibiting detectable PLCζ positively correlate with ICSI success rates (Yelumalai *et al.*, 2015). Thus, the clinical potential of PLCζ is apparent, both as a therapeutic intervention and as a prognostic indicator of OAD. One study (Meng *et al.*, 2020) recently further indicated the importance of examining PLCζ in the context of OAD by showing that 80% of patients exhibiting a significant ‘PLCζ deficiency’ who opted for AOA exhibited a significantly improved fertilization rate (∼40% higher) and improved pregnancy and live birth rates (both increased by 40% per initiated cycle).

Yoon *et al.* (2008) countered abnormalities in sperm PLCζ by co-injection with mouse PLCζ mRNA, while Rogers *et al.* (2004) demonstrated successfully generated parthenogenetic blastocysts following PLCζ cRNA injection into human oocytes. However, such therapeutic employment of PLCζ cRNA is not viable, as PLCζ transcription would be uncontrollable and extremely variable within oocytes, likely proving detrimental to pre-implantation development (Ducibella *et al.*, 2002; Rogers *et al.*, 2004; Ozil *et al.*, 2006). Consequently, the synthesis of a pure and active recombinant form of PLCζ has been a key goal (Kashir *et al.*, 2011; Nomikos *et al.*, 2013b), resulting in the generation of stable, purified recombinant human PLCζ protein, able to induce Ca^2+^ oscillations within a physiological range and able to counter the deleterious effects of mutant PLCζ (Nomikos *et al.*, 2013b). Furthermore, a mouse model of ICSI failure showed that success rates following PLCζ injection co-incident with such sperm were comparable to control injections (Sanusi *et al.*, 2015).

Importantly, the potential use of recombinant PLCζ as an AOA agent has one striking advantage; estimates of the quantity of PLCζ present in a single, healthy human sperm have been determined (50–100 fg/sperm), identifying a potential dosage range to examine (Kashir *et al.*, 2010; Nomikos *et al.*, 2013a; Kashir *et al.*, 2014; Saleh *et al.*, 2020). Furthermore, differences in PLCζ potency and Ca^2+^ oscillation patterns between humans and mouse models have been described (Heindryckx *et al.*, 2008; Nomikos *et al.*, 2013a; Swann and Lai, 2016; Swann, 2018, 2020), further facilitating a more accurate extrapolation of the effects of PLCζ on humans from animal models. Collectively, such studies underscore the potential for standardization of the dosage of recombinant PLCζ as an AOA activation agent and for overcoming the major obstacle of AOA activation agents.

However, despite PLCζ representing perhaps the most encouraging current physiological alternative to AOA with recombinant PLCζ still representing the most physiologically relevant AOA strategy, recent studies have necessitated a rethinking of what is currently accepted in terms of the physiological mechanism underlying mammalian oocyte activation. While both Hachem *et al.* (2017) and Nozawa *et al.* (2018) concluded that PLCζ was indeed the key driver of Ca^2+^ oscillations at least in mammals, indicating that sperm lacking PLCζ could not induce Ca^2+^ release following microinjection into mouse oocytes, IVF experiments with such sperm led to observations of Ca^2+^ oscillations, albeit lower in number and frequency, alongside a high degree of polyspermy and OAD (Nozawa *et al.*, 2018; Satouh and Ikawa, 2018). This abnormal pattern of Ca^2+^ release alongside low numbers of embryos and offspring, has been suggestively attributed towards spontaneous activation, unrelated to Ca^2+^ release, which is common in some strains of mice (Cheng *et al.*, 2012; Jones, 2018).

Another (perhaps more controversial) suggestion is that sperm potentially possess a secondary factor capable of Ca^2+^ releasing activity, albeit weaker than PLCζ (Jones, 2018; Swann, 2020). Proposed as an alternative ‘primitive’ or ‘cryptic’ sperm factor, some studies have suggested that this (yet unidentified) factor may also be involved in or contribute to events leading to oocyte activation (Nozawa *et al.*, 2018; Satouh and Ikawa, 2018; Swann, 2020). Alternatively, this cryptic factor could be one of the other potential candidates for the sperm factor apart from PLCζ as discussed previously herein, or perhaps PLCζ RNA may also be involved as the abnormal profile of Ca^2+^ release is very similar to injections of low RNA concentrations in mouse oocytes (Jones, 2018; Swann, 2020). However, as previously discussed, no other proposed factor has been independently and consistently confirmed to elicit physiological Ca^2+^ release, while the total amount of PLCζ RNA present within sperm may not be enough to elicit any Ca^2+^ at all (Jones, 2018; Swann, 2020). Collectively, this is an exciting and emerging area of investigation, and highlights just how much we do not know regarding the molecular intricacies involved in regulating Ca^2+^ release and oscillation patterns at fertilization.

### Further investigative avenues

Numerous further factors may contribute towards the efficacy of oocyte activation that could be utilized to replace current non-physiological mechanisms of oocyte activation, and thus require urgent further investigations. The mammalian oocyte machinery involved in Ca^2+^ release may also exert significant effects upon the efficacy of oocyte activation (Miyara *et al.*, 2003; Kilani and Chapman, 2014), with potential connections proposed between fertilization failure and the expression profiles of genes involved in oocyte maturation (Gasca *et al.*, 2008; Grøndahl *et al.*, 2013; Yeste *et al.*, 2016a, 2017). Such factors could also be playing at least a secondary role in the process of oocyte activation. Indeed, oocyte PLCs have often been suggested to play minor roles at least in the regulation of Ca^2+^ maintenance (Coward *et al.*, 2003; Runft *et al.*, 2004; Yeste *et al.*, 2016a).

Disruption of starfish egg cytoskeletal dynamics with heparin prevents the rapid Ca^2+^ wave upon interaction with sperm, instead delaying and reducing Ca^2+^ release and amplitude, failing to prevent polyspermy. This perhaps suggests that disruption of actin dynamics at fertilization influenced Ca^2+^ release (Puppo *et al.*, 2008; Santella *et al.*, 2015; Limatola *et al.*, 2019a), potentially also impacting upon subsequent events in egg activation such as cortical granule exocytosis (Santella *et al.*, 2015; Limatola *et al.*, 2019b). Thus, perhaps it is prudent to examine further preventative precautions to preserve actin cytoskeletal dynamics at fertilization as a part of AOA protocols. Furthermore, both direct PLCζ- and ICSI-induced mammalian oocyte activation generate zinc sparks, which coordinate with Ca^2+^ oscillations for embryos activated by PLCζ. These recent developments reveal the importance of fluctuations in zinc levels during activation (Tokuhiro and Dean, 2018; Que *et al.*, 2019). Accumulation of zinc throughout maturation seems essential for meiotic progression and meiotic arrest at metaphase II, and is then expelled from the oocyte at fertilization, immediately following the characteristic series of Ca^2+^ oscillations (Kim *et al.*, 2011a; Bernhardt *et al.*, 2012; Duncan *et al.*, 2016). Such dynamics of zinc appear to function by modulating CSF activity, in turn affecting maintenance of metaphase II arrest via modulating EMI2, a zinc-binding component of the CSF (Bernhardt *et al.*, 2012).

Indeed, zinc chelators in and of themselves prompted oocyte activation and blastocyst formation, lending themselves as possible additions to novel AOA protocols (Kerns *et al.*, 2018; Uh *et al.*, 2019). One such prospect is TPEN (Swann, 2018), a zinc chelator that enables meiotic resumption and embryogenesis in mice following ICSI using sperm in a mouse model of ICSI-failure (Suzuki *et al.*, 2010; Lee *et al.*, 2015). However, studies in humans have not yielded as promising results (Duncan *et al.*, 2016). In pig oocytes, TPEN is effective at causing activation, but only at lower concentrations and in combination with currently used Ca^2+^ ionophores (Lee *et al.*, 2015).

Further to direct processes, Ca^2+^-influx mechanisms are increasingly being revealed as indispensable aspects of oocyte activation, not only replenishing Ca^2+^ stores, but also underlying specific events such as polar body emission and cortical granule exocytosis. Defects in mediators of Ca^2+^ influx, including the TRPM7 and CaV3.2 channels, potentially alter the developmental potential of offspring by inducing a premature cessation of Ca^2+^ oscillations (Miao and Williams, 2012; Saleh *et al.*, 2020). Thus, perhaps mediation of such mechanisms represents an alternative method of treatment for cases of OAD and associated conditions of PLCζ-deficiency. Both the TRPM7 and Ca_V_3.2 channels almost completely account for Ca^2+^ influx in at least mammalian oocytes, while TRPM7 acts as a membrane sensor of extracellular magnesium (Mg^2+^) and Ca^2+^ concentrations, modulating the dynamics of the Ca^2+^ oscillatory response at fertilization (Miao and Williams, 2012; Miao *et al.*, 2012).

Indeed, culture conditions may also be another important contributory factor underlying successful utilization of AOA in the clinic. Patterns of Ca^2+^ release and subsequent embryogenesis were significantly affected by the concentration of Ca^2+^ in the culture media during AOA, indicating that the type of culture conditions used significantly exerts effects upon embryogenic competency in relation to Ca^2+^ release and embryogenesis (Lu *et al.*, 2018a). Mouse AOA with ionomycin in Ca^2+^-free medium for the 10 min duration of AOA yielded no blastocyst formation, despite subsequent culture of the embryos in media containing Ca^2+^ (Lu *et al.*, 2018a).

Altering the extracellular Mg^2+^ to Ca^2+^ ratio in culture media alters Ca^2+^ release dynamics in mouse oocytes at fertilization, as well as the developmental capacity of resultant embryos (Ozil *et al.*, 2017), suggesting that limiting Mg^2+^ availability in culture media may represent a potential intervention to increase Ca^2+^ release in cases where PLCζ-induced Ca^2+^ release may be defective via influx mechanisms including TRPM7 (Ozil *et al.*, 2017). However, focused clinical studies have not yet been undertaken to examine the overall effects of such alterations in culture media. It is essential to further examine whether modulation of Ca^2+^ influx mechanisms through alteration of culture media composition would improve pregnancy and delivery rates in the clinic, particularly in relation to PLCζ-associated conditions (Kashir, 2020).

## Future prospects

### Future of AOA on patients in the clinic

Despite various outstanding challenges to establishing the safety and efficacy of AOA, existing studies do not necessarily implicitly indicate that such methods are not promising or are unsafe. Importantly, most existing studies do not refer to the health of children born from AOA, but rather are endpoints during embryonic development that could potentially be remedied, depending of course on early detection and knowledge of potential problems in line with scientific advances to provide remedial solutions. For example, GM-CSF supplementation after AOA could reduce the occurrence of chromosomal abnormalities at *in*  *vitro* embryonic developmental during the fifth day (Economou *et al.*, 2017). Perhaps the main risk after AOA lies in a failure to coordinate the telophasic extrusion of the 2PB during MII, suggesting the adoption of chromosome screening to examine chromosomal ploidy (Capalbo *et al.*, 2016). Furthermore, embryo selection via the embryo grading system has yielded no further differences in pregnancy or implantation rates between AOA and standard ICSI groups (Aydinuraz *et al.*, 2016).

Importantly, as AOA is usually the final option for couples experiencing OAD, researchers have learned to utilize gametes in sub-optimal conditions, such as vitrified gametes or unfertilized oocytes post-ICSI (Liu *et al.*, 2011; Capalbo *et al.*, 2016; Economou *et al.*, 2017; Zhang *et al.*, 2017). Given that most ART protocols, including standard ICSI, prolonged *in*  *vitro* culture and cryopreservation, have been linked to altered gene expression (Ebner and Montag, 2016), hopes for the final safety and efficacy of AOA should be anticipated accordingly. The final clinical outcomes of AOA will likely be the result of the cumulative effect of all these procedures on the embryo, rather than just AOA alone. Therefore, adequate step-by-step screening for anomalies must be conducted to pinpoint exactly where and when AOA has the potential to cause harm. A thorough dissection of each technique’s molecular mechanism and affected pathways becomes all the more relevant. Still, it must be recognized that AOA remains the end of the line treatment option for an increasing number of patients, rendering this technique relevant and promising.

### Redesigning further studies

Further research on the efficacy and safety of AOA is required, particularly through larger scale studies. The establishment of an internationally recognized set of guidelines for AOA study design is of utmost importance. These include standardized protocols, clear criteria for patient inclusion and sub-classification, as well as a list of mandatory endpoints that can be universally used for efficacy measurement. As for safety assessments, the selection of a preferred set of tests, be it readily accessible DNA screening protocols, standardized tests or questionnaires, is recommended. Past studies indicate heterogeneity in clinical prediction models for ART (Ratna *et al.*, 2020). Additionally, multi-centre studies and international cooperation between different fertility centres would be preferred over single-centre studies to increase sample sizes and further promote protocol homogeneity. Finally, the establishment of a single, generalized database for AOA results could further orient and promote a standardized study design while facilitating direct comparison between studies. This would allow for quick protocol improvement, wide-spread acceptance of the technique and clinical establishment of AOA.

In contrast, combinations and variations of experimental techniques should only be explored if and when standardized protocols prove insufficient or unsafe. Furthermore, clear and direct technique efficacy comparisons should be made across studies, as has been done by precedent studies (Tesarik *et al.*, 2002; Kyono *et al.*, 2012; Nikiforaki *et al.*, 2016; Economou *et al.*, 2017). Nonetheless, the exact molecular mechanisms of each ART are still unclear in humans, further complicating the choice of the best protocol for each situation.

Finally, it is vital that current research on the safety, efficacy and efficiency of ARTs, including AOA, is clearly conveyed to patients, who might otherwise take it upon themselves to seek information online about infertility treatment options. Many freely available resources do not predominantly rely upon academic studies, yet they may sway public opinion on AOA. Hence, the public availability of clear and informative studies about AOA could crucially influence public awareness, create a greater sense clinician-patient trust and help patients make informed, autonomous decisions about their own treatment plans. On balance, AOA appears an immensely promising avenue of treatment for not just OAD and TFF, but perhaps even to improve the efficacy of ART overall. However, the identification of more physiological agents and protocols that better mimic physiological Ca^2+^ release is essential, coupled with further studies to evaluate the safety and efficacy of such treatments, before such procedures can be widely applied within clinics.

## Data availability

No new data were generated or analysed in support of this research.

## Authors’ roles

J.K. and D.G. contributed towards most of the literature search, quality assessment, data extraction and interpretation of results, alongside C.J. and K.C. J.K., D.G., C.J. and K.C. were all involved in drafting of the manuscript, and all authors approved of the final version of the article.

## Funding

D.G. was supported by Stanford University’s Bing Overseas Study Program. J.K. was supported by a Healthcare Research Fellowship Award (HF-14-16) from Health and Care Research Wales (HCRW), alongside a National Science, Technology, and Innovation plan (NSTIP) project grant (15-MED4186-20) awarded by the King Abdulaziz City for Science and Technology (KACST).

## Conflict of interest

The authors have no conflicts of interest to declare.
